# New sulfonamide-based glycosides incorporated 1,2,3-triazole as cytotoxic agents through VEGFR-2 and carbonic anhydrase inhibitory activity

**DOI:** 10.1038/s41598-024-62864-9

**Published:** 2024-06-06

**Authors:** Hebat-Allah S. Abbas, Eman S. Nossier, May A. El-Manawaty, Mohamed N. El-Bayaa

**Affiliations:** 1 Department of Photochemistry, National Research Centre, Cairo, 12622 Egypt; 2 Department of Pharmaceutical Medicinal Chemistry and Drug Design Department, Faculty of Pharmacy (Girls), Al-Azhar University, Cairo, 11754 Egypt; 3 The National Committee of Drugs, Academy of Scientific Research and Technology, Cairo, 11516 Egypt; 4 Pharmacognosy Department, Pharmaceutical and Drug Industries Research Institute, National Research Centre, Cairo, 12622 Egypt; 5 Department of Chemistry, College of Science, Qassim University, 51452 Buraidah, Saudi Arabia

**Keywords:** Sulfonamide-based, 1,2,3-Triazole, Copper, Antiproliferative, VEGFR-2, Carbonic anhydrase, Cancer, Chemistry, Organic chemistry

## Abstract

New sulfonamide-triazole-glycoside hybrids derivatives were designed, synthesised, and investigated for anticancer efficacy. The target glycosides’ cytotoxic activity was studied with a panel of human cancer cell lines. Sulfonamide-based derivatives, **4**, **7** and **9** exhibited promising activity against HepG-2 and MCF-7 (IC_50_ = 8.39–16.90 μM against HepG-2 and 19.57–21.15 μM against MCF-7) comparing with doxorubicin (IC_50_ = 13.76 ± 0.45, 17.44 ± 0.46 μM against HepG-2 and MCF-7, rescpectively). To detect the probable action mechanism, the inhibitory activity of these targets was studied against VEGFR-2, carbonic anhydrase isoforms hCA IX and hCA XII. Compoumds **7** and **9** gave favorable potency (IC_50_ = 1.33, 0.38 μM against VEGFR-2, 66, 40 nM against hCA IX and 7.6, 3.2 nM against hCA XII, respectively), comparing with sorafenib and SLC-0111 (IC_50_ = 0.43 μM, 53 and 4.8 nM, respectively). Moreover, the docking simulation was assessed to supply better rationalization and gain insight into the binding affinity between the promising derivatives and their targeted enzymes that was used for further modification in the anticancer field.

## Introduction

Cancer is a horrible affliction that has plagued humans for centuries, causing unspeakable suffering and loss. As a serious disease, it kills about 7 million people worldwide each year. By 2025, the population is anticipated to increase to 19.3 million people^1,2^. Anticancer medications can successfully eradicate cancer cells, but they frequently have significant side effects as well, like hair loss, nausea, genotoxicity, weakened immune system efficiency, and drug resistance^3–6^. Research is currently being done to create novel approaches that focus on signaling pathways^7,8^. Compared to chemotherapy, targeted therapy has demonstrated fewer hazardous adverse effects through targeting signaling pathways unique to cancer^9^.

Vascular endothelial cells are the exclusive target of the positive regulator vascular endothelial growth factor (VEGF). The primary modulator of VEGF-induced responses in endothelial cells, VEGFR-2 is a tyrosine kinase subtype that can regulate microvascular permeability, proliferation, and differentiation. Several malignancies, such as hepatocellular carcinoma, melanoma, thyroid, ovarian, colorectal, and breast cancers, as well as medulloblastomas, overexpress VEGFR-2^10–17^. Recently, it was revealed that some drugs with antiangiogenic activity or powerful inhibitors of VEGFR-2 in vitro had been utilized successfully in clinical trials to treat cancer^18^.

All organisms contain carbonic anhydrases (CAs), a superfamily of metalloenzymes with an active center that contain a metallic Zn^2+^ ion^19,20^. Catalyzing the reversible conversion of carbon dioxide into bicarbonate is the primary function of carbonic anhydrase in metabolism^21–23^. Mammals are the source of sixteen distinct CA isoforms, each of them plays a vital physiological role. A variety of them are membrane-bound (CA IV, CA IX, CA XII, CA XIV, and CA XV), some are cytosolic (CA I, CA II, CA III, CA VII, and CA XIII), mitochondrial (CA VA and CA VB), and secreted in milk and saliva (CA VI)^24,25^.

As a result of the hypoxic environment that cancer progression develops, extracellular hypoxic tumors become more acidic, which accelerates growth and metastasis of tumor^26^. Transmembrane hCA IX regulates extracellular and intracellular pH under hypoxic conditions, which is linked to the invasion and advancement of various malignancies, including brain, breast, lung, cervical, neck, colon and bladder cancers^27^. Also, hypoxic tumours typically exhibit increased resistance to conventional anticancer therapy as well as increased tumour aggression^28^. Consequently, hCA IX is presently viewed as a desirable anticancer target. Moreover, hCA XII is frequently overexpressed in aggressive tumours^29^. By limiting angiogenesis and metastasis, selective inhibition of hCA IX and XII activity has been shown to slow tumour growth and impact its ability to spread^30^. Therfore, targeting these isoenzymes in anticancer drug designs is essential due to their expression in hypoxic tumor cells^31^.

Sulfonamides are molecules with a wide range of biological activities that can potentially applied as possible therapeutic agents in drug development and discovery. Sulfonamides have wide applications as HIV protease inhibitors, antiviral^32^, antibacterial^33,34^, anti-inflammatory^35^, and anticancer drugs^36–40^. It is also widely known that certain sulfonamide derivatives have antimetabolite properties^41^. Additionally, it has been demonstrated that sulfonamide moieties combined with various heterocyclic ingredients impair the proliferation of human cancer cell lines especially breast cells^42,43^. Triazoles have emerged as key components in medicinal chemistry because of their many pharmacological applications like anticancer, antioxidant, antiviral, antitubercular, antibacterial, and anti-inflammatory activities^44^. Moreover, 1,2,3-triazoles have been discovered to be resistant to metabolic degradation and their presence in molecules may support an increase in their solubility and bioavailability, which advocate their utilization as pharmacophoric moieties^45^.

**Figure 1:**
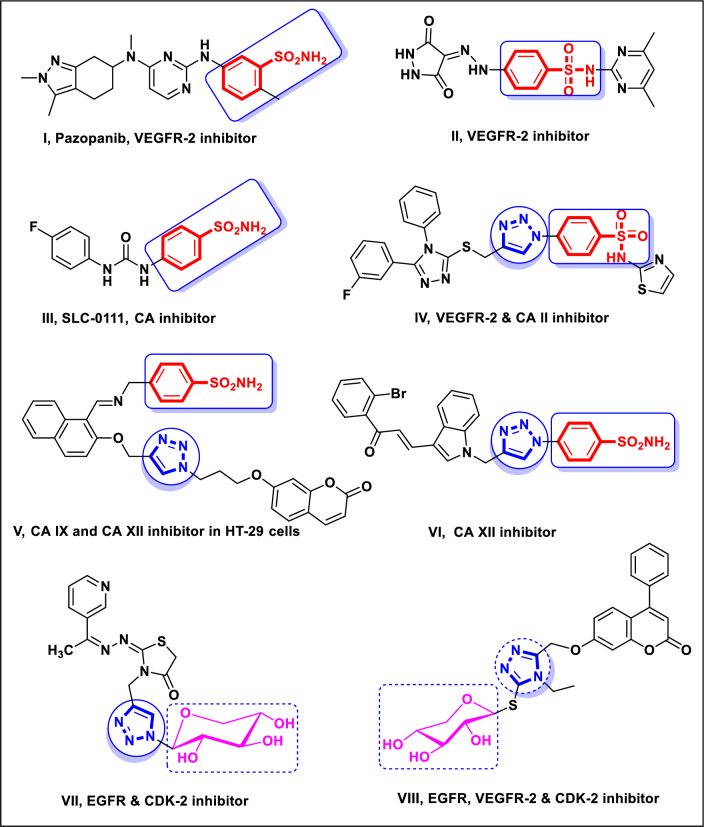
Cytotoxic agents with different mechanistic approaches that include benzenesulfonamide, 1,2,3-triazole, and/or glycoside scaffolds.

Potent VEGFR-2 inhibitor pazopanib **I**, which has sulfonamide moiety, has been developed and approved for the remediation of many types of cancer^16^. In 2021, Sayed et al.^46^ found that sulfonamide derivative **II** combined with a 3,5-dioxopyrazolidine scaffold was highly cytotoxic through its inhibitory activity against VEGFR-2 (Fig. 1). SLC-0111, a ureido benzenesulfonamide molecule **III**, appears to be in Phase I/II clinical studies for advanced metastatic solid malignancies^47^ and exerts its action through inhibition of carbonic anhydrase isoforms IX and XII. Incorporating benzenesulfonamide with 1,2,3-triazole scaffold in **IV**–**VI** exhibited considerable in vitro cytotoxicity against several human cancer cell lines by inhibiting VEGFR-2 and/or carbonic anhydrase activities^48–50^ (Fig. 1). However, the addition of glycosides to heterocyclic compounds has generated significant hybrids with biologically valuable characteristics, particularly antiviral, anticancer, and antibacterial properties^51^. Compounds with triazole-glycoside motifs **VII** and **VIII** have demonstrated substantial cytotoxic and inhibitory impacts on EGFR, CDK-2, and/or VEGFR-2^52,53^ (Fig. 1).

**Figure 2:**
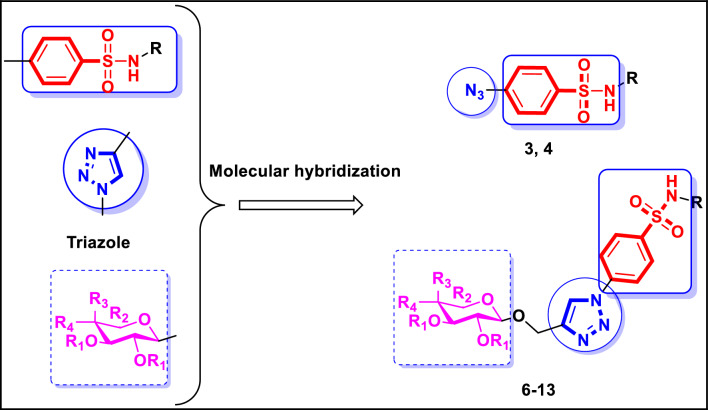
Design route of benzenesulfonamide-based targets bearing azido group in 3, 4 or 1,2,3-triazole-glycoside scaffolds in 6–13 targeting VEGFR-2 and Carbonic anhydrase inhibition.

Considering the achievements of the past and our ongoing desire to identify efficacious chemotherapeutic targets^54–57^, a novel series of benzenesulfonamide-based targets bearing an azido group in **3**,** 4** or 1,2,3-triazole-glycoside scaffolds in **6**–**13** were were designed and synthesized through molecular hybridization of the three crucial cores: benzenesulfonamide, 1,2,3-triazole, and glycoside (Fig. 2). All synthesized compounds based on benzenesulfonamide were evaluated against cancer cell lines of the lung (A-549), liver (HepG-2), breast (MCF-7), and colon (HCT-116) To determine their action mechanism, the remarkable compounds were then assessed for their inhibitory potential against VEGFR-2 and the carbonic anhydrase isoforms hCA IX and hCA XII. They were additionally examined for their impact on the cell cycle, apoptosis, p-53, and the apoptotic proteins Bax and Bcl-2. Finally, the binding views within the chosen enzymes’ active sites were predicted using in silico docking simulation.

## Results and discussion

### Chemistry

**Scheme 1:**
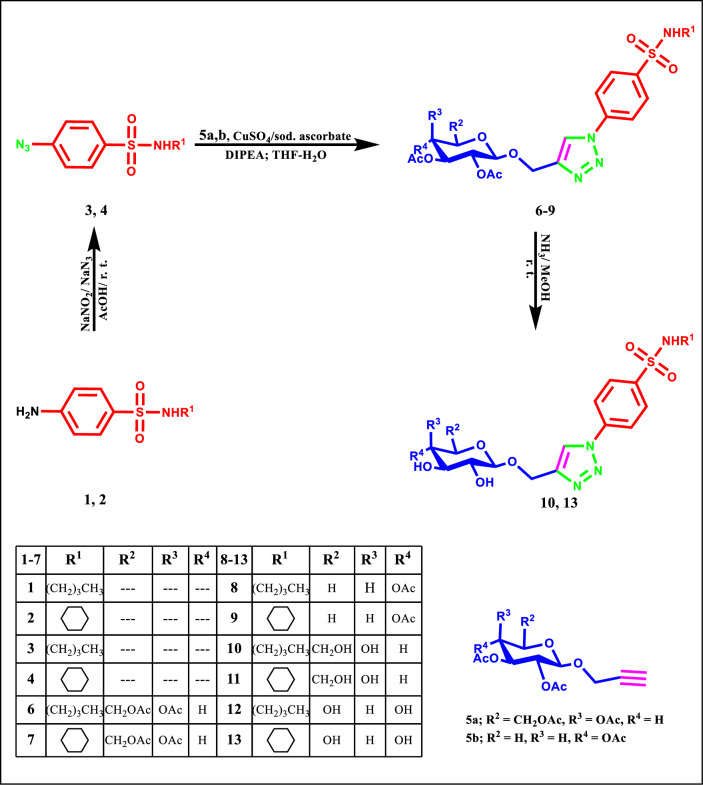
Synthesis of 1,2,3-triazolyl hybrid glycosides.

The CuAAC reaction is known as copper-catalyzed cycloaddition of azides with terminal alkynes yields 1,4-di-substituted 1,2,3-triazoles and it was first reported in 2002 by Tornøe et al.^58^ and Rostovtsev et al.^59^. In the present work, the synthesis of the designed target compounds benzene sulfonamides (**6**–**13**) containing triazole *C*-glycoside tails were achieved by Cu-catalyzed 1,3-DCR of the azido scaffold **3** and** 4** with a panel of acylated *O*-propynyl glycosides **5a**,**b** (Scheme1).

The synthetic route started with the synthesis of 4-azido-benzenesulfonamide derivatives **3** and** 4** through the reaction of *N*-butylsulfanilamide **1** or *N*-cyclohexylsulfanilamide **2**^60^ with sodium azide. The IR spectrum of 4-azido derivatives **3** and **4** includes the disappearance of the absorption band for NH_2_ and the appearance of new bands (2101 and 2100) cm^-1^ due to the formation of azide group respectively.

The synthesized azide derivatives **3** and** 4** were reacted with the propargylated glycosides that; galacto, xylo-pyranosyl compound **5a**,**b**, under click conditions by Cu-catalyzed cycloadition reaction (CuAAC) to achieve the 1,2,3-triazole glycosides derivatives **6–9** (Scheme 1). The required Cu(I) catalyst was generated by adding Na-ascorbate and copper sulfate which converted from Cu(II) in situ reaction at the medium to afford the targeted 1,2,3-triazole products. In the structures of the last glycosides, the sugar moiety was linked to C^4^ of the triazole system, as analogs of modified *C*-nucleosides. The IR spectra of compounds **6**–**9** proved the characteristic bands related to (C=O) frequency in the acetate group. Their ^1^H NMR spectra demonstrated the signals special to the sugar fragments which was appeared to the formed *β*-confirmation of the triazole-sugar linkage as proved by the coupling constant of the anomeric proton. The acetylated glycosides **6**–**9** derivatives was deprotected under the medium of a saturated ammonia solution in methanol and afforded the free hydroxyl triazole glycosides **10**–**13**, which was approved with their spectral data. Disappearing of the acetyl-carbonyl absorption bands and appearing of the hydroxyl groups of the afforded deacetylated glycosidic compounds at their characteristic regions proved the structure. Finally, the NMR spectra signified the deactylation reaction and showed the existing of the hydroxyl protons signals and the disappearance of the signals of the methyl protons of the acetyl groups.

### In vitro cytotoxic activity

**Figure 3:**
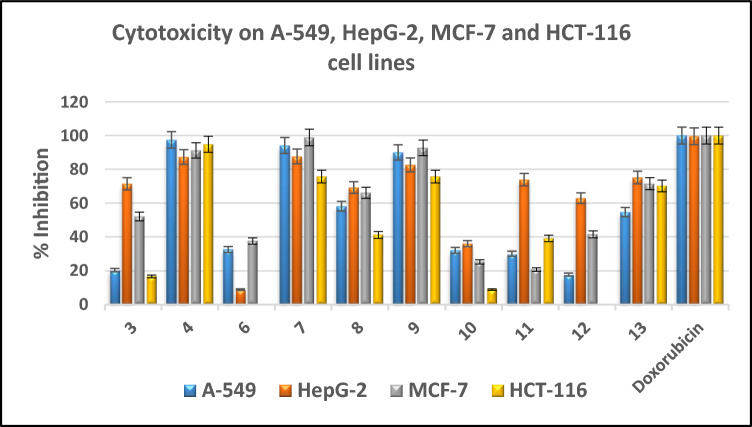
Preliminary cytotoxic evaluation of sulfonamide-based targets 3, 4, 6–13 through the MTT assay against human cancerous A-549, HepG-2, MCF-7, HCT-116 cell lines at 100 µM comparing with doxorubicin.

Using lung A-549, liver HepG-2, breast MCF-7 and colorectal HCT-116 cancer cell lines, preliminary cytotoxic efficacy of sulfonamide-based derivatives **3, 4, 6**–**13** was illustrated in vitro through MTT assay^61–63^ at a concentration of 100 μM comparing with doxorubicin and sunitinib (Fig. 3, Table S1). Promising derivatives exhibiting cytotoxic potency of at least 60% at 100 μM were further assessed in comparison to their respective cells at several concentrations ranging from (100–12.5 μM) in Tables S2–S5, in order to ascertain their IC_50_, as presented in Table 1. The sulfonamide derivatives **3**, **8**, **12** and **13** afforded from moderate to weak cytotoxicity against all screened cell lines, however the derivatives **4**, **7** and **9** exhibited promising activity against HepG-2 and MCF-7 (IC_50 range_ = 8.39–16.90 μM against HepG-2 and 19.57–21.15 μM against MCF-7) comparing with doxorubicin (IC_50_ = 13.76 ± 0.45, 17.44 ± 0.46 μM against HepG-2 and MCF-7, rescpectively). The latter derivatives furnished moderate cytotoxicity against A-549 and HCT-116 (IC_50 range_ = 19.81–30.91 μM against A-549 and 20.30–32.05 μM against HCT-116) regarding to sunitinib (IC_50_ = 10.14 ± 0.50, 9.67 ± 0.22 μM against A-549 and HCT-116, rescpectively). Also, compound **11** gave promising activity against HepG-2 (IC_50_ = 16.90 ± 0.09 μM) with weak ones against the rest cell lines.

**Table 1:** The antitumor activities of the target compounds against cancerous A-549, HepG-2, MCF-7, HCT-116 and normal RPE-1 cell lines expressed as IC_50_ values.

Compd. No	IC_50_ (mean ± SD) (µM)				
	A-549	HepG-2	MCF-7	HCT-116	RPE-1
3	–	57.56 ± 12.23	–	–	–
4	30.91 ± 0.65	12.53 ± 0.51	19.57 ± 1.10	20.30 ± 0.33	78.27 ± 1.56
7	20.45 ± 0.28	10.45 ± 0.13	20.31 ± 0.66	32.05 ± 0.42	82.58 ± 0.52
8	–	47.02 ± 0.51	46.49 ± 0.42	–	–
9	19.81 ± 0.65	8.39 ± 0.20	21.15 ± 2.45	23.60 ± 0.22	87.22 ± 0.73
11	–	16.90 ± 0.09	–	–	–
12	–	36.44 ± 0.12	–	–	–
13	–	30.01 ± 0.06	30.83 ± 0.20	53.07 ± 0.69	–
Doxorubicin	–	13.76 ± 0.45	17.44 ± 0.46		–
Sunitinib	10.14 ± 0.50			9.67 ± 0.22	

*IC*_50_ Compound concentration required to inhibit growth by 50%, *SD* Standard deviation, each value is the mean of three values, (–) not detected.

Furthermore, the MTT assay was utilized to estimate the cytotoxic activity of the extremely potent derivatives **4**, **7** and **9** against the normal cell line RPE-1, with the aim of examining their safety profiles (Table 1). The IC_50_ values of these compounds were higher, ranging from 78.27 ± 1.56 to 87.22 ± 0.73 µM. They could therefore be thought of as safe cytotoxic agents.

### Structure activity relationship study

**Figure 4:**
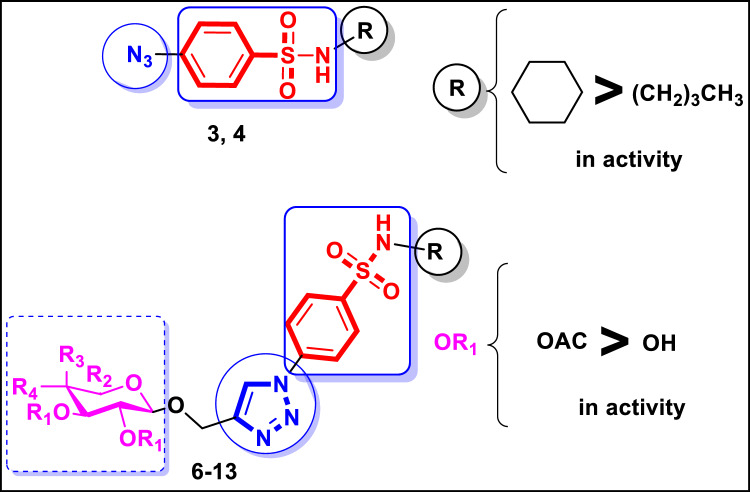
SAR study of sulfonamide-based derivatives 3, 4, 6–13 as cytotoxic agents against human cancer A-549, HepG-2, MCF-7, HCT-116 cell lines.

According to previous results shown in Table 1, it was noted that the azido derivatives of sulfonamide incorporating with *N*-butylbenzene in **3**, displayed weak cytotoxicity against all screened cell lines. In contrast, substitution with *N*-cyclohexylbenzene in **4**, exhibited moderate activity against A-549 and HCT-116 (IC_50_ = 30.91 ± 0.65 and 20.30 ± 0.33 μM, respectively) and promising potency against HepG-2 and MCF-7 (IC_50_ = 12.53 ± 0.51 and 19.57 ± 1.10 μM, respectively). Replacement of azido group with 1,2,3-triazole-glycoside hybrids in **6**–**9** afforded variable activities against tested cell lines ranging from weak to promising ones. In case of acetylated glycosides, the promising cytotoxicity was observed from derivatives 7 and 9 bearing *N*-cyclohexylbenzene against HepG-2 and MCF-7 (IC_50_ = 10.45, 8.39 μM against HepG-2 and 20.31, 21.15 μM against MCF-7, respectively), and moderate activity against the rest cell lines A-549 and HCT-116 (IC_50_ = 20.45, 19.81 μM against A-549 and 32.05, 23.60 μM against HCT-116, respectively). On the other hand, hydroxylated glycosides **10**–**13**, revealed weak cytotoxicity except *N*-cyclohexylbenzene derivatives **11**, **13** expressing promising activity against HepG-2 (IC_50_ = 16.90 and 30.01 μM, respectively). Moreover, the hydroxylated glycoside **13** gave moderate activity against MCF-7 (IC_50_ = 30.83 ± 0.20 μM) (Fig. 4).

In summary, sulfonamide-based hybrids bearing *N*-cyclohexylbenzene incorporated with azido fragment or 1,2,3-triazole linked to acetylated glycosides, exhibited promising potency against the screened cancerous cell lines HepG-2 and MCF-7.

### In vitro enzyme inhibitory assessment against VEGFR-2 and Carbonic anhydrase isoforms hCA IX and hCA XII

Aiming to elucidate action mechanism, the sulfonamide-based targets **4, 7** and **9** were selected for were selected for further evaluation of their in vitro inhibitory potency against VEGFR-2 and the carbonic anhydrase isoforms hCA IX and hCA XII due to their outstanding cytotoxic results. Their IC_50_ values were recorded in Table 1 using sorafenib and SLC-0111 as references, respectively^64,65^. 

As descriped in Table 2, the sulfonamide-based target bearing azido group **4** revealed weak inhibitory activity against all screened enzymes comparing. On the other hand, 1,2,3-triazole-glycoside hybrids **7** and **9** gave promising potency with higher selectivity of **9** than **7** against VEGFR-2, hCA IX and hCA XII (IC_50_ = 1.33 μM, 66 and 7.6 nM, respectively for **7**, 0.38 μM, 40 and 3.2 nM, respectively for **9**) comparing with sorafenib and SLC-0111 (IC_50_ = 0.43 μM, 53 and 4.8 nM, respectively).

**Table 2:** In vitro Inhibitory assessment of the promising sulfonamide-based derivatives 4, 7 and 9 against VEGFR-2 and carbonic anhydrase isoforms hCA IX and hCA XII comparing with sorafenib and SLC-0111, respectively.

Compound No	IC_50_ (mean ± SD)		
	VEGFR-2 (µM)	Carbonic anhydrase isoforms (nM)	
		hCA IX	hCA XII
4	181 ± 0.66	142	35.9
7	1.33 ± 0.10	66	7.6
9	0.38 ± 0.14	40	3.2
Sorafenib	0.43 ± 0.10	–	–
SLC-0111	–	53	4.8

*IC*_50_ Compound concentration necessary to inhibit the enzyme activity by 50%, *SD* Standard deviation, each value is the mean of three values, (–) not detected.

Previous study suggested that sulfonamide-based derivatives with 1,2,3-triazole-glycoside fragments **7** and **9** inhibited VEGFR-2, hCA IX and hCA XII activities, resulting in antiproliferative potency.

### Cell cycle arrest and apoptosis of compound 9

**Figure 5:**
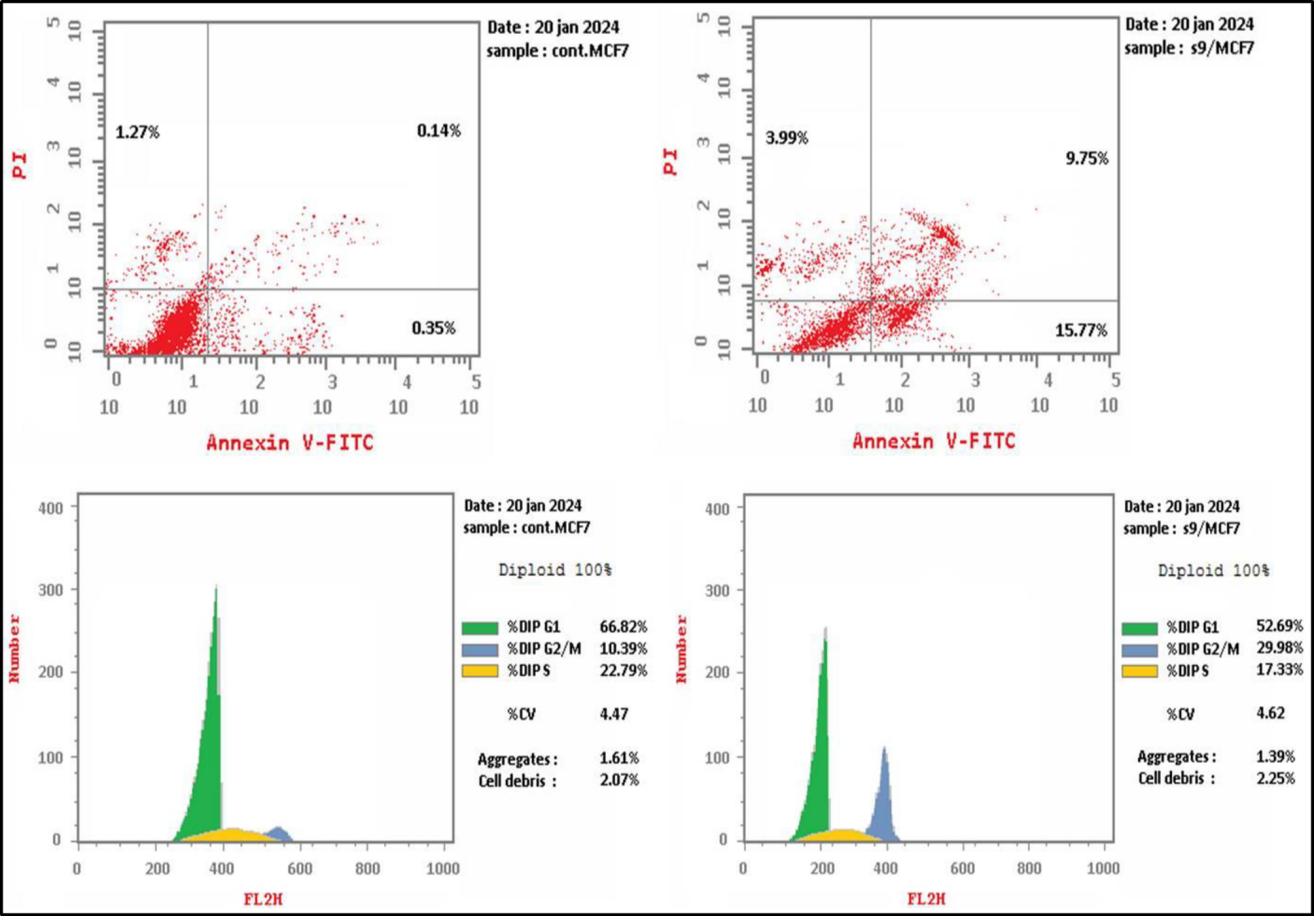
Examination of cell cycle and influence of benzenesulfonamide-1,2,3-triazole-glycoside 9 on the percentage of V-FITC-positive annexin staining in MCF-7 cells regarding to control.

**Figure 6:**
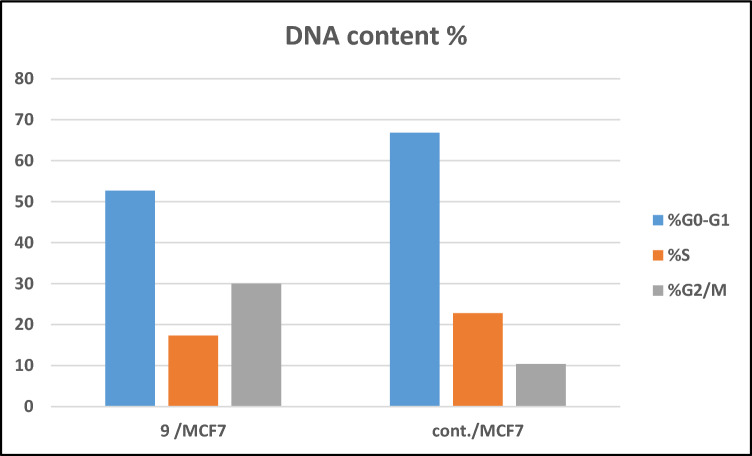
Examination of cell cycle with benzenesulfonamide-1,2,3-triazole-glycoside 9.

**Figure 7:**
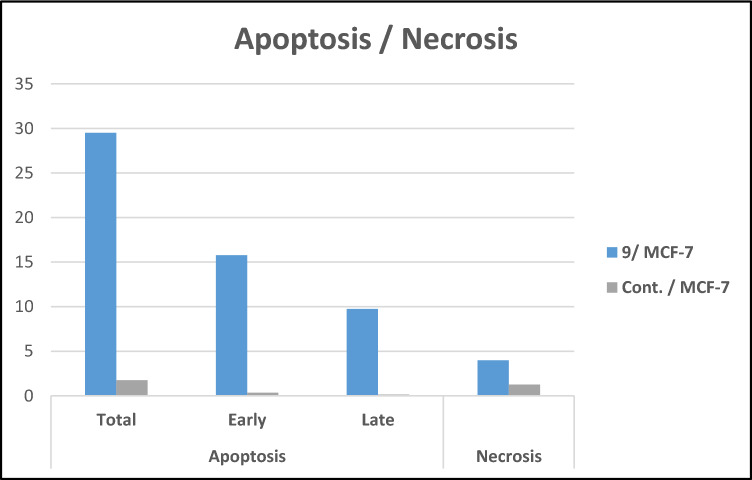
Influence of benzenesulfonamide-1,2,3-triazole-glycoside 9 on apoptotic activity.

MCF-7 cells were treated for 24 h at a concentration of 21.15 μM to assess if the most potent cytotoxic derivative, benzenesulfonamide-1,2,3-triazole-glycoside **9**, causes cell death using the apoptotic mechanism. The cells were further examined through flow cytometry with annexin-V staining (Figs. 5–7).

Target 9 demonstrated higher cell accumulations of 29.98% during the G2/M phase than untreated MCF-7 cells, which indicated 10.39%, as shown in Fig. 6 and Table S6. The data obtained clearly illustrated that derivative **9** is able to arrest MCF-7 cells in the G2/M phase of the cell cycle.

The investigated benzenesulfonamide-1,2,3-triazole-glycoside **9** resulted in a significant increase to 15.77% in the early apoptosis from 0.35% (DMSO control) and a noticeable increase to 9.75% in the late apoptosis from 0.14% (DMSO control) with regard to apoptosis (Fig. 7 and Table S7). Furthermore, the derivative produced a 3.99% necrosis percentage as opposed to the DMSO control’s 1.27%. Consequently, compound **9** may induce apoptosis, as suggested through a notable boost in apoptotic cells.

### Impact of benzenesulfonamide-1,2,3-triazole-glycoside 9 upon Bax, Bcl-2 and p53 levels in MCF-7 cells

The two main mechanisms that control the cell during apoptosis are the extrinsic pathway, which is mediated by the death receptor, and the intrinsic pathway, which is mediated by the mitochondria^66^. The roles of Bcl-2 as an anti-apoptotic and Bax as a pro-apoptotic (inducer) allow the two proteins to modify this programmed process, and the balance between them regulates cell death^67^. The tumor suppressor gene, p53, is an additional essential component that results in cell death or inhibits cell proliferation. Cancers that maintain their genomic stability and p53 inhibition may increased cell proliferation and become resistant to numerous anticancer treatments^68^.

MCF-7 cells treated for 24 h with compound **9**’s IC_50_ of 21.15 μM showed a 6.2-fold increase in Bax levels (271.45 Pg/mL) compared to untreated control cells (43.66 Pg/mL).On the other hand, Bcl-2 protein was downregulated by 2.6 times in MCF-7 cells treated with compound **9**, going from 8.51 to 3.27 ng/mL. Additionally, compound **9** boosted the p53 protein level by 7.4 times in MCF-7 treated cells compared to 125.40 Pg/mL in control cells (Table 3).

**Table 3:** Impact of the benzenesulfonamide-1,2,3-triazole-glycosides 9 upon Bax, Bcl-2 and p53 levels.

Compd	Bax (Pg/mL)	Bcl-2 ng/mL	Bax/Bcl-2	P53 (Pg/mL)
9/MCF-7	271.45 ± 0.25	3.27 ± 0.18	83.01	930.69 ± 0.55
Cont./MCF-7	43.66 ± 1.30	8.51 ± 0.26	5.13	125.40 ± 0.15

### Molecular docking simulation

Docking simulation of benzenesulfonamide-1,2,3-triazole-glycosides **7** and **9** against VEGFR-2 and the carbonic anhydrase isoforms hCA IX and hCA XII was accomplished to establish a relationship through their activities and potential binding modes within the binding sites of the evaluated enzymes, based on the promising results obtained from the in vitro inhibitory assessment.

The docking processes were performed using the MOE-Dock (Molecular Operating Environment) software version 2014.0901^69,70^. Initially, the procedures were verified through re-docking of the native ligands, sorafenib and acetazolamide, within the active sites of VEGFR-2, hCA IX and hCA XII (PDB codes: 4ASD, 3IAI and 1JG0, respectively)^64,71,72^. This resulted in energy score values of − 10.73, − 9.88 and − 9.65 kcal/mol with comparatively small values of RMSD (0.85, 1.22 and 1.16 Å, respectively), between the native ligands and their docked positions.

**Figure 8:**
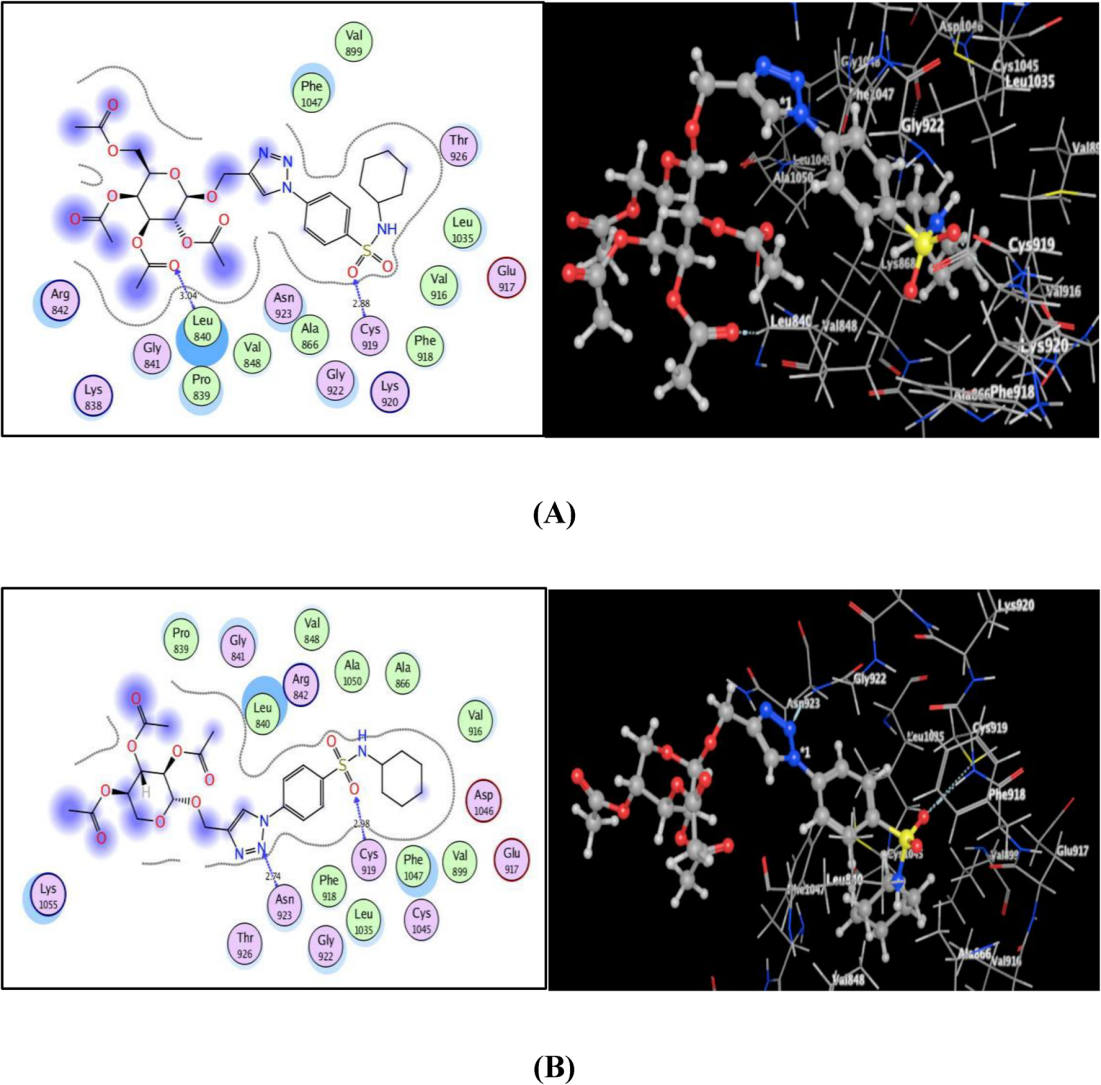
(**A**) & (**B**) views illustrated (2D and 3D) binding features of the benzenesulfonamide 1,2,3-triazole glycosides 7 and 9 within the active site of VEGFR 2 (PDB code: 4ASD), respectively.

When compared to the original ligand, sorafenib, the screened 1,2,3-triazole-glycosides **7** and **9** displayed promising binding inside the VEGFR-2 active site, as shown in Fig. 8, with noted energy score values of − 9.45 and − 10.73 kcal/mol. The sulfonamide oxygen of both derivatives **7** and **9** afforded H-bond acceptors with the backbone of the key amino acid **Cys919** (distance: 2.88 and 2.98 Å, respectively), resembling the parent ligand, sorafenib. Furthermore the acetyl oxygen of glycoside part in **7** formed H-bond acceptor with the backbone of **Leu840** (distance: 3.04 Å). Removal of acetylmethyl fragment at p-6 of glycoside part in **9**, pushed it away from **Leu840** facilitated binding of triazole nitrogen with the backbone of **Asn923** through H-bonding (distance: 2.74 Å).

**Figure 9:**
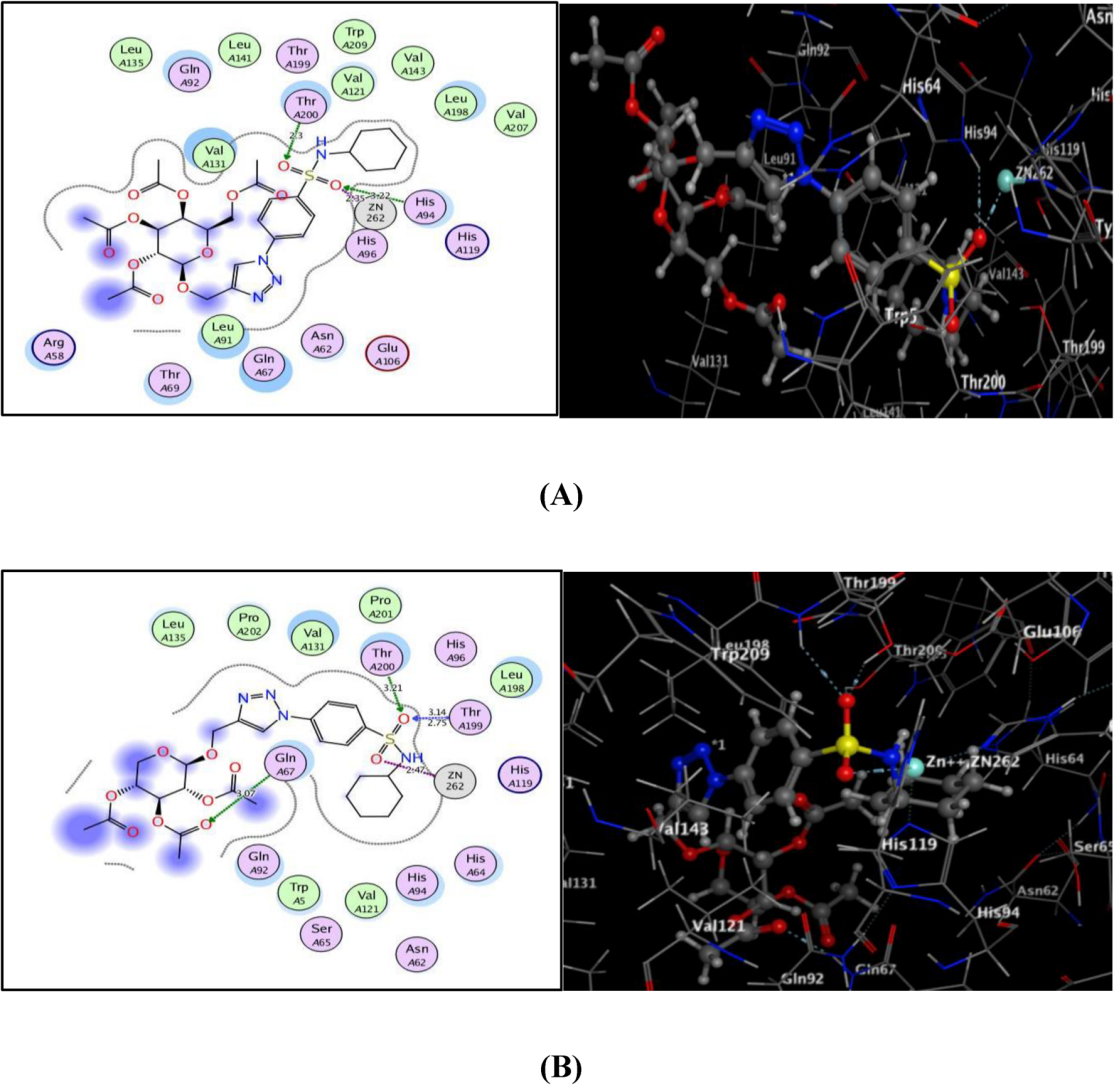
(**A**) & (**B**) views illustrated (2D and 3D) binding features of the benzenesulfonamide 1,2,3-triazole glycosides 7 and 9 within the active site of hCA IX (PDB code: 3IAI), respectively.

**Figure 10:**
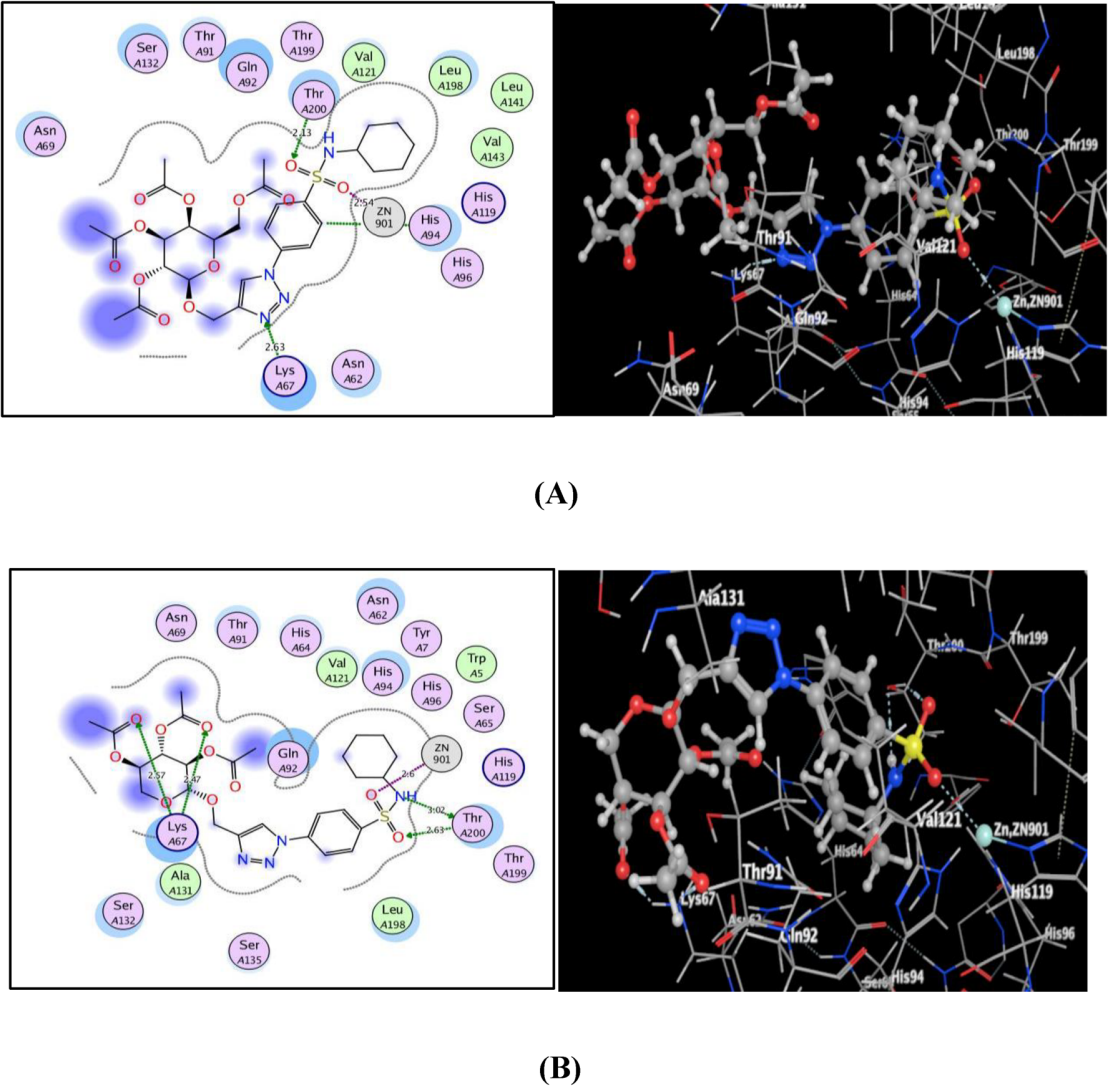
(**A**) & (**B**) views illustrated (2D and 3D) binding features of the benzenesulfonamide-1,2,3-triazole-glycosides 7 and 9 within the active site of hCA XII (PDB code: 1JD0), respectively.

Regarding to carbonic anhydrase isoforms hCA IX and hCA XII in Figs. 9 and 10, the sufonamide moiety in both **7** and **9** strengthened the binding within their active sites through generation of H-bond acceptor with the sidechain of **Thr200** and ionic bond with Zinc ion. Additionally, the sidechain of **Gln67** revealed H-bonding with acetyl oxygen in **9** within hCA IX and hCA XII, and with triazole nitrogen in **7** within hCA XII. Sulfonamide oxygen gave one H-bonding with **His94** in **7** and two with **Thr199** in **9** within hCA IX (distance: 3.22, 2.75 and 3.14 Å, respectively).

The incorporation of 1,2,3-triazole glycosides **7** and **9** with sulfonamide moiety provided significant fixing through several bonds inside the binding sites of VEGFR-2, hCA IX, and hCA XII. Through additional hydrophobic and hydrophilic interactions, the absence of the acetylmethyl fragment at position 6 of the glycoside portion in **9** encouraged its fitting with the active sites of the screening enzymes rather than its counterpart **7**.

## Conclusion

New derivatives of benzenesulfonamide class based on carbohydrates were designed by a sugar-tail approach and efficiently synthesized. The anticancer activity results against lung A-549, liver HepG-2, MCF-7 and colorectal HCT-116 cancer cell lines detect high potency of sulfonamide-based targets **4**, **7** and **9** against HepG-2 and MCF-7 cell lines (IC_50 range_ = 8.39–16.90 μM against HepG-2 and 19.57–21.15 μM against MCF-7), in comparison with doxorubicin as a reference drug. These compounds were in vitro studied against VEGFR-2, carbonicanhydrase isoforms hCA IX and hCA XII yielding promising broad inhibitory activity of *N*-cyclohexylbenzenesulfonamide-1,2,3-triazole-glycosides **7** and **9** (IC_50_ = 1.33, 0.38 μM against VEGFR-2, 66, 40 nM against hCA IX and 7.6, 3.2 nM against hCA XII, respectively), comparing with references, sorafenib and SLC-0111 (IC_50_ = 0.43 μM, 53 and 4.8 nM, respectively).. The molecular docking studies proved promising enzymatic results and summarized good binding patterns and well-fitting behavior of compounds **7** and **9** in the active sites of VEGFR-2, hCA IX, and hCA XII.

## Experimental section

### General experimental methods

Reichert Thermovar apparatus was used to recording the melting points which was uncorrected. Perkin-Elmer model 1720 FTIR spectrometer was used to recording the Infra-red spectra. Bruker AC-300 or DPX-300 spectrometer (500 MHz ^1^H) (125 MHz ^13^C) was used to investigate the Nuclear magnetic resonance. The values of δ_ppm_ (chemical shifts) were registered compared to TMS as a standard reference. The coupling constants (J values) are offered in Hz. TLC holding aluminum silica gel 60 F245 was used to checking The advance of reaction completion. IR, ^1^H NMR, ^13^C NMR, Elemental analyses were measured at the National Research Center, Egypt.

### Synthesis of azid 3 and 4 derivatives

Sulfanilamide derivatives **1** and **2** (0.007 mol) was dissolved in conc. acetic acid (18 mL) and distilled water (18 mL), then cooled in an ice bath until reach the temperature to (0–5) °C, then sodium nitrite solution (0.47 g, 0.007 mol in (5 mL) water) was added very slowly to the mixture and stirred for (10 min.). Finally, the solution of sodium azide (0.45 g, 0.007 mol in (25 mL) distilled water) was added drop-by-drop to the reaction mixture and stirred for (30 min.). The solid product was filtered off and washed with distilled water to obtain compounds **3** and** 4**. Copmpound **3** was previously synthesized^73^.

### 4-Azido-N-cyclohexylbenzenesulfonamide (4)

Yield: 85%; m.p. 65–67 °C; IR (KBr) cm^−1^, *ν*: 3223 (NH), 2890 (CH-aliph.), 2100 (N=N=N), 1337, 1168 (SO_2_). ^1^H-NMR (500 MHz, DMSO-d_6_) δ/ppm: 8.78 (d, 2H,* J* = 8.6 Hz, Ar–H), 7.55 (br. s, 1H, NH), 7.31 (d, 2H,* J* = 8.6 Hz, Ar–H), 2.72–2.68 (m, 1H, N–CH_2_), 1.34–1.31 (m, 2H, CH_2_), 1.25–1.21 (m, 2H, CH_2_), 1.19–1.16 (m, 4H, 2CH_2_), 0.86–0.84 (m, 2H, CH_2_). ^13^C-NMR (125 MHz, DMSO-d_6_) δ/ppm: 143.4, 136.9, 128.5, 119.7, 42.4, 31.3, 28.9, 25.9, 22.1, 13.9. Analysis calcd. for C_12_H_16_N_4_O_2_S (280.35): C, 51.41; H, 5.75; N, 19.99. Found: C, 51.49; H, 5.65; N, 20.04.%.

### Click reaction 6–9 derivatives

To a well stirred mixture of the acetylated acetylenic glacto, xylo-pyranosyl sugar (3 mmol) and the azido benzenesulfonamide derivatives **3** and** 4** (2 mmol) in THF*–*H_2_O (2:1; 25 mL) was added CuSO_4_·5H_2_O (0.4 mmol) then add quickly Na-ascorbate (0.4 mmol) and 3–4 drops of diisopropylethylamine at 0 °C. The reaction mixture was stirred at 60 °C for 12 h (TLC, petroleum ether–EtOAc, 4:1). the organic layer was separated after adding ethyl acetate (30 mL), washed with water (2 × 40 mL) and dried. The residue was purified by column chromatography (petroleum ether—EtOAc, 4:1) to obtain the acetylated glycosyl triazole derivatives **6**–**9**.

### N-butyl-4-(4-([2,3,4,6-tetra-O-acetyl-β-D-galactopyranosyl]oxymethyl)-1H-1,2,3-triazol-1-yl)benzenesulfonamide (6)

Yield: 77%; m.p. yellowish foam; IR (KBr) cm^−1^, *ν*: 3203 (NH), 2907 (CH-aliph.), 1740 (C=O), 1330, 1158 (SO_2_). ^1^H-NMR (500 MHz, CDCl_3_) δ/ppm: 8.13 (s, 1H, triazole), 8.03 (d, 2H,* J* = 7.5 Hz, Ar–H), 7.92 (d,1H,* J* = 8.3 Hz, Ar–H), 7.83 (d, 1H,* J* = 8.3 Hz, Ar–H), 7.26 (br. s, 1H, NH), 6.36 (d, 1H,* J* = 2.4 Hz, H-1′), 5.49 (t, 1H,* J* = 2.2 Hz, H-3′), 5.41 (d, 1H, *J* = 3.2 Hz, H-2′), 5.32 (s, 2H, CH_2_), 5.05 (dd, 1H,* J* = 10.6, 3.6 Hz, H-4′), 4.33 (d, 1H,* J* = 6.6 Hz, H-5′), 4.06–4.11 (m, 2H, H-6′′,H-6*’*), 2.99 (t, 2H,* J* = 7.0 Hz, CH_2_), 2.15, 2.03, 2.01, 1.99, (4 s, 12H, CH_3_CO), 1.44–1.50 (m, 2H, CH_2_), 1.28–1.32 (m, 2H, CH_2_), 0.86 (t, 3H, *J* = 7.2 Hz, CH_3_). ^13^C NMR (125 MHz, CDCl_3_) δ 170.2, 169.9, 169.8, 168.9, 140.3, 128.9, 120.5, 119.3, 100.4, 89.4, 70.6, 68.6, 67.3, 67.2, 66.3, 61.1, 42.8, 31.3, 20.6, 20.5, 20.4, 20.3, 19.5, 13.3. Analysis calcd. for C_27_H_36_N_4_O_12_S (640.66): C, 50.62; H, 5.66; N, 8.75. Found: C, 50.54; H, 5.73; N, 8.65.%.

### N-cyclohexyl-4-(4-([2,3,4,6-tetra-O-acetyl-β-D-galactopyranosyl]oxymethyl)-1H-1,2,3-triazol-1-yl)benzenesulfonamide (7)

Yield: 75%; m.p. yellowish foam; IR (KBr) cm^−1^, *ν*: 3225 (NH), 2881 (CH-aliph.), 1750 (C=O), 1340, 1170 (SO_2_). ^1^H-NMR (500 MHz, CDCl_3_) δ/ppm: 8.16 (s, 1H, triazole), 8.02 (d, 2H,* J* = 8.3 Hz, Ar–H), 7.91 (d, 2H,* J* = 8.2 Hz, Ar–H), 7.26 (br. s, 1H, NH), 6.36 (d, 1H,* J* = 2.5 Hz, H-1′), 5.48 (t, 1H,* J* = 1.9 Hz, H-3′), 5.40 (d, 1H, *J* = 2.6 Hz, H-2′), 5.31 (s, 2H, CH_2_), 5.06–5.03 (m, 1H, H-4′), 4.33 (d, 1H,* J* = 6.7 Hz, H-5′), 4.09–4.06 (m, 2H, H-6′′,H-6*’*), 2.98–2.96 (m, 1H, N–CH), 2.14, 2.02, 2.00, 1.99, (4 s, 12H, CH_3_CO), 1.48–1.46 (m, 2H, CH_2_), 1.26–1.20 (m, 6H, 3CH_2_), 0.86–0.82 (m, 2H, CH_2_).^13^C NMR (125 MHz, CDCl_3_) δ 170.3, 170.1, 169.8, 168.9, 140.4, 128.9, 120.5, 100.5, 89.6, 70.7, 68.7, 67.8, 67.4, 66.4, 61.2, 43.2, 31.7, 29.3, 26.4, 22.5, 20.8, 20.6, 20.5, 20.4, 13.9. Analysis calcd. for C_29_H_38_N_4_O_12_S (666.70): C, 52.25; H, 5.75; N, 8.40. Found: C, 52.36; H, 5.80; N, 8.31.%.

### N-butyl-4-(4-([2,3,4-tri-O-acetyl-β-D-xylopyranosyl]oxymethyl)-1H-1,2,3-triazol-1-yl)benzenesulfonamide (8)

Yield: 80%; m.p. yellowish foam; IR (KBr) cm^−1^, *ν*: 3208 (NH), 2900 (CH-aliph.), 1737 (C=O), 1331, 1155 (SO_2_). ^1^H-NMR (500 MHz, CDCl_3_) δ/ppm: 8.19 (s, 1H, triazole), 8.02–7.92 (m, 4H, Ar–H), 7.27 (br. s, 1H, NH), 5.18 (d, 1H, H-1′), 5.09–4.99 (m, 2H, H-2′, H-3′), 4.96 (s, 2H, CH_2_), 4.73–4.71 (m, 1H, H-4′), 4.34–4.18 (m, 1H, H-5′), 3.85–3.41 (m, 1H, H-5′′), 2.98 (t, 2H, CH_2_), 2.07, 2.04, 2.01, (3 s, 9H, CH_3_CO), 1.47–1.28 (m, 4H, CH_2_), 0.85 (t, 3H, *J* = 8.1 Hz, CH_3_). ^13^C NMR (125 MHz, CDCl_3_) δ 13.4, 19.6, 20.6, 20.7, 25.5, 29.6, 31.5, 42.9, 55.7, 62.1, 62.3, 67.8, 68.8, 70.8, 71.3, 98.3, 100.1, 120.5, 128.5, 128.9, 139.5, 140.4, 169.7, 169.9, 170.1. Analysis calcd. for C_24_H_32_N_4_O_10_S (568.60): C, 50.70; H, 5.67; N, 9.85. Found: C, 50.66; H, 5.73; N, 9.92.%.

### N-cyclohexyl-4-(4-([2,3,4-tri-O-acetyl-β-D-xylopyranosyl]oxymethyl)-1H-1,2,3-triazol-1-yl)benzenesulfonamide (9)

Yield: 79%; m.p. yellowish foam; IR (KBr) cm^−1^, *ν*: 3244 (NH), 2894 (CH-aliph.), 1756 (C=O), 1341, 1173 (SO_2_). ^1^H-NMR (500 MHz, CDCl_3_) δ/ppm: 8.13 (s, 1H, triazole), 8.04 (d, 2H,* J* = 7.7 Hz, Ar–H), 7.93 (d, 2H,* J* = 7.7 Hz, Ar–H), 7.25 (br. s, 1H, NH), 5.18 (d, 1H,* J* = 4.0 Hz, H-1′), 5.03–5.01 (m, 1H, H-3′), 4.95 (s, 2H, CH_2_), 4.75–4.69 (m, 1H, H-2′), 4.32 (d, 1H,* J* = 3.3 Hz, H-4′), 4.18–4.11 (m, 1H, H-5′), 3.44–3.39 (m, 1H, H-5′′), 3.00–2.97 (m, 1H, N–CH), 2.05, 2.04, 2.03 (3 s, 9H, CH_3_CO), 1.50–1.45 (m, 2H, CH_2_), 1.25–1.21 (m, 6H, 3CH_2_), 0.87–0.84 (m, 2H, CH_2_).^13^C NMR (125 MHz, CDCl_3_) δ 170.1, 169.7, 129.1, 120.8, 100.3, 98.4, 75.4, 71.3, 70.9, 70.5, 68.9, 62.1, 55.7, 43.5, 31.9, 29.7, 29.5, 29.1, 26.6, 22.7, 20.8, 14.1. Analysis calcd. for C_26_H_34_N_4_O_10_S (594.64): C, 52.52; H, 5.76; N, 9.42. Found: C, 52.43; H, 5.69; N, 9.50.%.

### Synthesis of deacetylated O-glycosides (10–13)

This reaction was achieved by adding a saturated methanolic ammonia solution (15 mL) at 0 °C for 20 min. to the acetylated 1,2,3-triazole glycoside **6**–**9** which dissolved in methanol. then stirried at room temperature for 8 h., until the completion of the deacetylation process (TLC, petroleum ether–hexane, 2:1), By using the rotatory, the solvent was evaporated under reduced pressure at 40 °C to obtain a yellowish powder residue, which crystallized from ethanol to yield compound **10**–**13**.

### N-butyl-4-(4-([β-D-Galactopyranosyl]oxymethyl)-1H-1,2,3-triazol-1-yl)benzenesulfonamide (10)

Yield: 60%; m.p. 100–102 °C; IR (KBr) cm^−1^, *ν*: 3455–3425 (OH), 3182 (NH), 2917 (CH-aliph.), 1331, 1159 (SO_2_). ^1^H-NMR (500 MHz, DMSO-*D*_6_) δ/ppm: 8.95 (s, 1H, triazole), 8.14 (d, 2H, Ar–H), 8.03–7.98 (m, 2H, Ar–H), 7.73 (br. s, 1H, NH), 5.37 (d, 1H, H-1′), 4.94–4.92 (m, 1H, OH), 4.78–4.73 (m, 1H, OH), 4.28 (s, 2H, CH_2_), 3.82–3.78 (m, 1H, OH), 3.69–3.65 (m, 1H, 1OH), 3.56–3.55 (m, 2H, H-3′, H-2′), 3.46 (d, 1H, H-4′), 3.42–3.38 (m, 2H, H-6*’*, H-5′), 3.36–3.35 (m, 1H, H-6′′), 2.78–2.63 (m, 2H, CH_2_), 1.83–1.75 (m, 2H, CH_2_), 1.37–1.21 (m, 2H, CH_2_), 0.79 (t, 3H, CH_3_).^13^C NMR (125 MHz, DMSO-*D*_6_) δ 145.7, 140.1, 139.1, 128.5, 122.9, 120.5, 103.1, 75.5, 73.5, 70.7, 68.4, 61.4, 60.7, 42.3, 31.1, 19.2, 13.5. Analysis calcd. for C_19_H_28_N_4_O_8_S (472.51): C, 48.30; H, 5.97; N, 11.86. Found: C, 48.21; H, 6.06; N, 11.91.%.

### N-cyclohexyl-4-(4-([β-D-galactopyranosyl]oxymethyl)-1H-1,2,3-triazol-1-yl)benzenesulfonamide (11)

Yield: 59%; m.p. 118–120 °C; IR (KBr) cm^−1^, *ν*: 3451–3435 (OH), 3187 (NH), 2927 (CH-aliph.), 1333, 1158 (SO_2_). ^1^H-NMR (500 MHz, DMSO-*D*_6_) δ/ppm: 8.94 (s, 1H, triazole), 8.16–8.12 (m, 2H, Ar–H), 8.02–7.98 (m, 2H, Ar–H), 7.72 (br. s, 1H, NH), 5.37 (d, 1H, H-1′), 4.91 (s, 2H, CH_2_), 4.77–4.73 (m, 1H, OH), 4.68–4.62 (m, 1H, OH), 4.44–4.37 (m, 1H, OH), 4.28–4.27 (m, 1H, 1OH), 3.98–3.90 (m, 1H, H-2*’*), 3.81–3.76 (m, 1H, H-3′), 3.71–3.64 (m, 2H, H-5*’*, H-4′), 3.57–3.54 (m, 1H, H-6*’*), 3.46–3.45 (m, 1H, H-6′′), 2.79–2.76 (m, 1H, N–CH), 1.78–1.75 (m, 2H, CH_2_), 1.37–1.34 (m, 2H, CH_2_), 1.25–1.22 (m, 2H, CH_2_), 1.18–1.16 (m, 2H, CH_2_), 0.84–0.82 (m, 2H, CH_2_). ^13^C NMR (125 MHz, DMSO-*D*_6_) δ 145.7, 140.5, 139.1, 128.5, 122.8, 120.4, 103.1, 75.5, 73.5, 70.7, 68.4, 61.4, 60.7, 42.6, 31.3, 28.9, 26.1, 22.1, 13.9. Analysis calcd. for C_21_H_30_N_4_O_8_S (498.55): C, 50.59; H, 6.07; N, 11.24. Found: C, 50.67; H, 5.99; N, 11.29.%.

### N-butyl-4-(4-([β-D-Xylopyranosyl]oxymethyl)-1H-1,2,3-triazol-1-yl)benzenesulfonamide (12).

Yield: 55%; m.p. 150–152 °C; IR (KBr) cm^−1^, *ν*: 3449–3439 (OH), 3183 (NH), 2932 (CH-aliph.), 1346, 1174 (SO_2_). ^1^H-NMR (500 MHz, DMSO-*D*_6_) δ/ppm: 8.93 (s, 1H, triazole), 8.14 (d, 2H,* J* = 8.3 Hz, Ar–H), 7.99 (d, 2H,* J* = 8.4 Hz, Ar–H), 7.72 (br. s, 1H, NH), 5.06 (d, 1H,* J* = 5.1 Hz, H-1′), 4.99 (s, 2H, CH_2_), 4.90–4.87 (m, 1H, OH), 4.72–4.70 (m, 1H, OH), 4.31–4.29 (m, 1H, 1OH), 3.77–3.74 (m, 1H, H-2′), 3.47–3.42 (m, 1H, H-3′), 3.14–3.08 (m, 2H, H-5*’*, H-4′), 3.03–3.01 (m, 1H, H-5′′), 2.80–2.77 (m, 2H, CH_2_), 1.38–1.33 (m, 2H, CH_2_), 1.27–1.21 (m, 2H, CH_2_), 0.79 (t, 3H, CH_3_). ^13^C NMR (125 MHz, DMSO-*D*_6_) δ 145.4, 140.4, 138.9, 128.4, 122.7, 120.5, 102.9, 76.6, 73.3, 69.6, 65.8, 61.2, 42.3, 31.1, 19.2, 13.4. Analysis calcd. for C_18_H_26_N_4_O_7_S (442.49): C, 48.86; H, 5.92; N, 12.66. Found: C, 48.78; H, 6.00; N, 12.59.%.

### N-cyclohexyl-4-(4-([β-D-Xylopyranosyl]oxymethyl)-1H-1,2,3-triazol-1-yl)benzenesulfonamide (13)

Yield: 58%; m.p. 160–162 °C; IR (KBr) cm^−1^, *ν*: 3442–3436 (OH), 3185 (NH), 2922 (CH-aliph.), 1335, 1168 (SO_2_). ^1^H-NMR (500 MHz, DMSO-*D*_6_) δ/ppm: 8.93 (s, 1H, triazole),8.14 (d, 2H,* J* = 8.3 Hz, Ar–H), 7.99 (d, 2H,* J* = 8.5 Hz, Ar–H), 7.71 (br. s, 1H, NH), 5.07 (d, 1H,* J* = 5.0 Hz, H-1′), 4.99 (s, 2H, CH_2_), 4.90–4.87 (m, 1H, OH), 4.72–4.70 (m, 1H, OH), 4.31–4.29 (m, 1H, 1OH), 3.78–3.74 (m, 1H, H-2′), 3.32–3.29 (m, 1H, H-3′), 3.14–3.08 (m, 2H, H-5*’*, H-4′), 3.04–3.00 (m, 1H, H-5′′), 2.79–2.76 (m, 1H, N–CH), 1.38–1.34 (m, 2H, CH_2_), 1.24–1.16 (m, 6H, 3CH_2_), 0.81–0.84 (m, 2H, CH_2_). ^13^C NMR (125 MHz, DMSO-*D*_6_) δ 145.4, 140.5, 139.1, 128.4, 122.5, 120.4, 102.9, 76.6, 73.3, 69.6, 65.8, 61.3, 42.6, 31.3, 28.9, 26.1, 22.1, 13.9. Analysis calcd. for C_20_H_28_N_4_O_7_S (468.53): C, 51.27; H, 6.02; N, 11.96. Found: C, 51.19; H, 5.92; N, 12.03.%.

### Cytotoxic activity

The newly synthesised sulfonamide-based derivatives **3, 4** and** 6**–**13** were estimated for their cytotoxic activities in vitro using the MTT assay method, following the published procedure^61–63^, using human lung A-549, liver HepG-2, breast MCF-7, colorectal HCT-116 cancer cell lines, and human retinal pigment epithelial normal RPE-1 cell line. The supplemental file contained extra details.

### Inhibitory assessment against VEGFR-2 and carbonic anhydrase isoforms hCA IX and hCA XII activities

The promising sulfonamide-based derivatives **4, 7** and **9** were assessed in vitro for their inhibitory effect against VEGFR-2, hCA IX, and hCA XII activities, in complying with the previously outlined technique, using sorafenib and SLC-0111 as references^64,65^. There was further information in the supplemental file.

### Detection of cell cycle analysis and apoptosis of compound 9

The examination of cell cycle analysis and apoptosis was explained^66^ with the use of flow cytometry. Benzenesulfonamide-1,2,3-triazole-glycosides **9** was applied to MCF-7 cells for a duration of 24 h, and the cells were then incubated at 37 °C. Further details were included in the supplemental file.

### The effect of compound 9 on MCF-7 cell levels of Bax, Bcl-2 and p53

Bax, Bcl-2 and p53 levels in MCF-7 cells were clarified utilising the previously reported approach for the promising benzenesulfonamide-1,2,3-triazole-glycosides **9**.

### Molecular docking simulation

The rationalization of biological discovers has become easier with the assistance of computational docking simulation. The promising benzenesulfonamide-1,2,3-triazole-glycosides **7** and **9** were docked inside the active sites of VEGFR-2 and the carbonic anhydrase isoforms hCA IX and hCA XII (PDB codes: 4ASD, 3IAI, and 1JG0, respectively)^64,71,72^ through the use of MOE-Dock (Molecular Operating Environment) software version 2014.0901^69,70^. Full descriptions are available in the supplementary material.

### Supplementary Information

Supplementary Information.
