# Supplementary material for: New sulfonamide-based glycosides incorporated 1,2,3-triazole as cytotoxic agents through VEGFR-2 and carbonic anhydrase inhibitory activity
Source: Sci Rep. 2024 Jun 6;14:13028. doi: 10.1038/s41598-024-62864-9 (PMC11156913; doi:10.1038/s41598-024-62864-9)
Supplement: Supplementary file 1 — Supplementary Information. [file 41598_2024_62864_MOESM1_ESM.docx]

**New Sulfonamide-Based Glycosides Incorporated 1,2,3-Triazole as Cytotoxic Agents through VEGFR-2 and Carbonic Anhydrase Inhibitory Activity**

**Hebat-Allah S. Abbas^1*^, Eman, S. Nossier^2,3^, May A. El-Manawaty^4^, Mohamed N. El-Bayaa^1,5^**

^1^Department of Photochemistry, National Research Centre, Cairo 12622, Egypt.

^2^Department of Pharmaceutical Medicinal Chemistry and Drug Design Department, Faculty of Pharmacy (Girls), Al-Azhar University, Cairo, 11754, Egypt.

^3^The National Committee of Drugs, Academy of Scientific Research and Technology, Cairo, 11516, Egypt.

^4^Pharmacognosy Department, Pharmaceutical and Drug Industries Research Institute, National Research Centre, Cairo 12622, Egypt.

^5^Department of Chemistry, College of Science, Qassim University, Buraidah 51452, Saudi Arabia.

**Supplementary material**

1. **Experimental**
   1. **Chemistry**

**4-Azido-*N*-butylbenzenesulfonamide (3):**

Yield: 88%; m.p. 47–49 °C; IR (KBr) cm^−1^, *ν*: 3203 (NH), 2907 (CH-aliph.), 2101 (N=N=N), 1330, 1158 (SO_2_). ^1^H-NMR (500 MHz, DMSO-d_6_) δ/ppm: 7.79 (d, 2H, *J* = 8.6 Hz, Ar-H), 7.56 (br. s, 1H, NH), 7.31 (d, 2H, *J* = 8.6 Hz, Ar-H), 2.71 (t, 2H, *J* = 7.0 Hz, CH_2_), 1.38-1.28 (m, 2H, CH_2_), 1.26-1.17 (m, 2H, CH_2_), 0.79 (t, 3H, *J* = 7.3 Hz, CH_3_). ^13^C-NMR (125 MHz, DMSO-d_6_) δ/ppm: 144.1, 137.5, 129.1, 120.2, 42.7, 31.6, 19.7, 13.9. Analysis calcd. for C_10_H_14_N_4_O_2_S (254.31): C, 47.23; H, 5.55; N, 22.03. Found: C, 47.18; H, 6.01; N, 21.93.%.

**
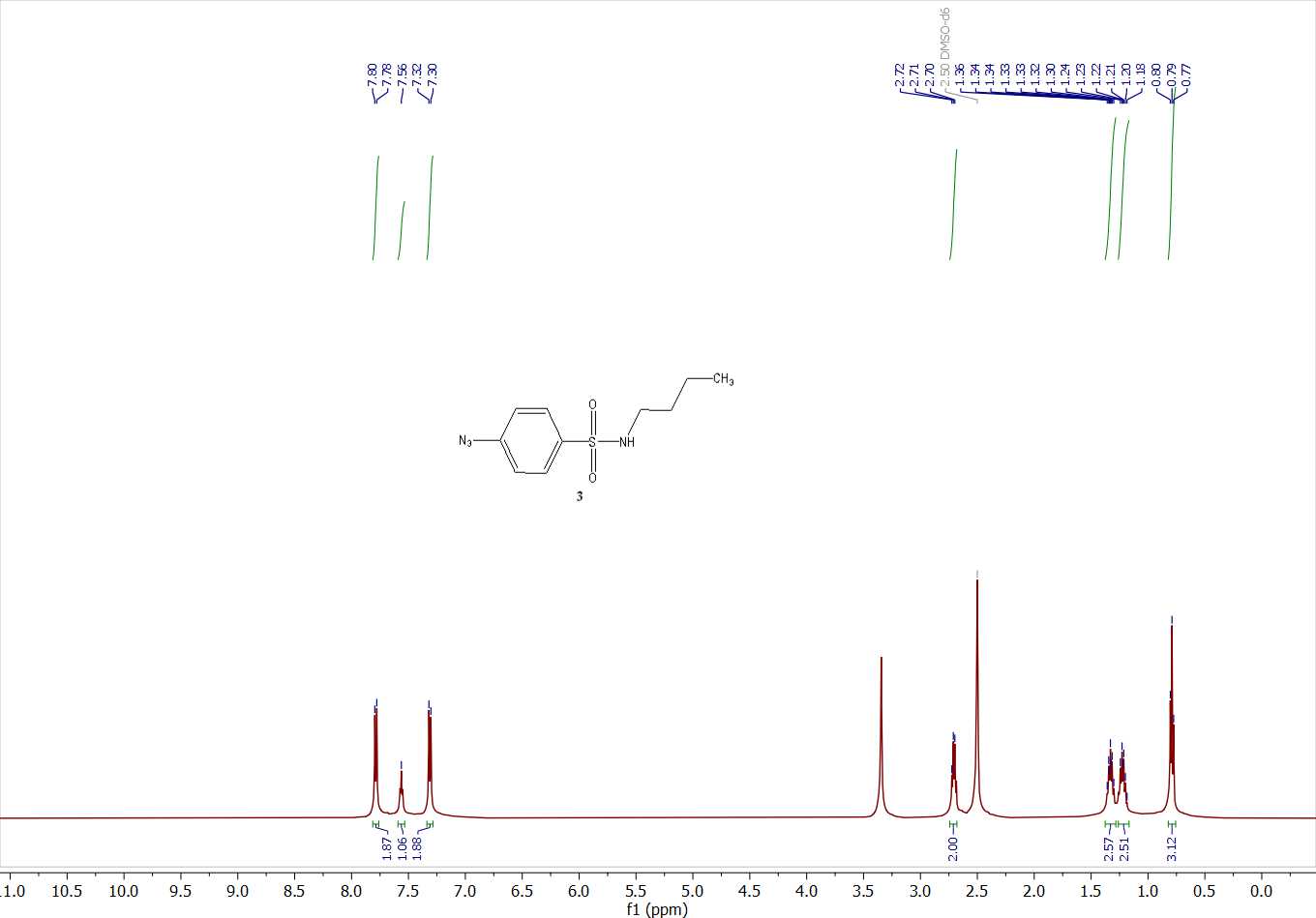
**

Figure S1. ^1^H NMR spectrum of compound **3** (DMSO-d_6_, 500 MHz, 298 K).

**
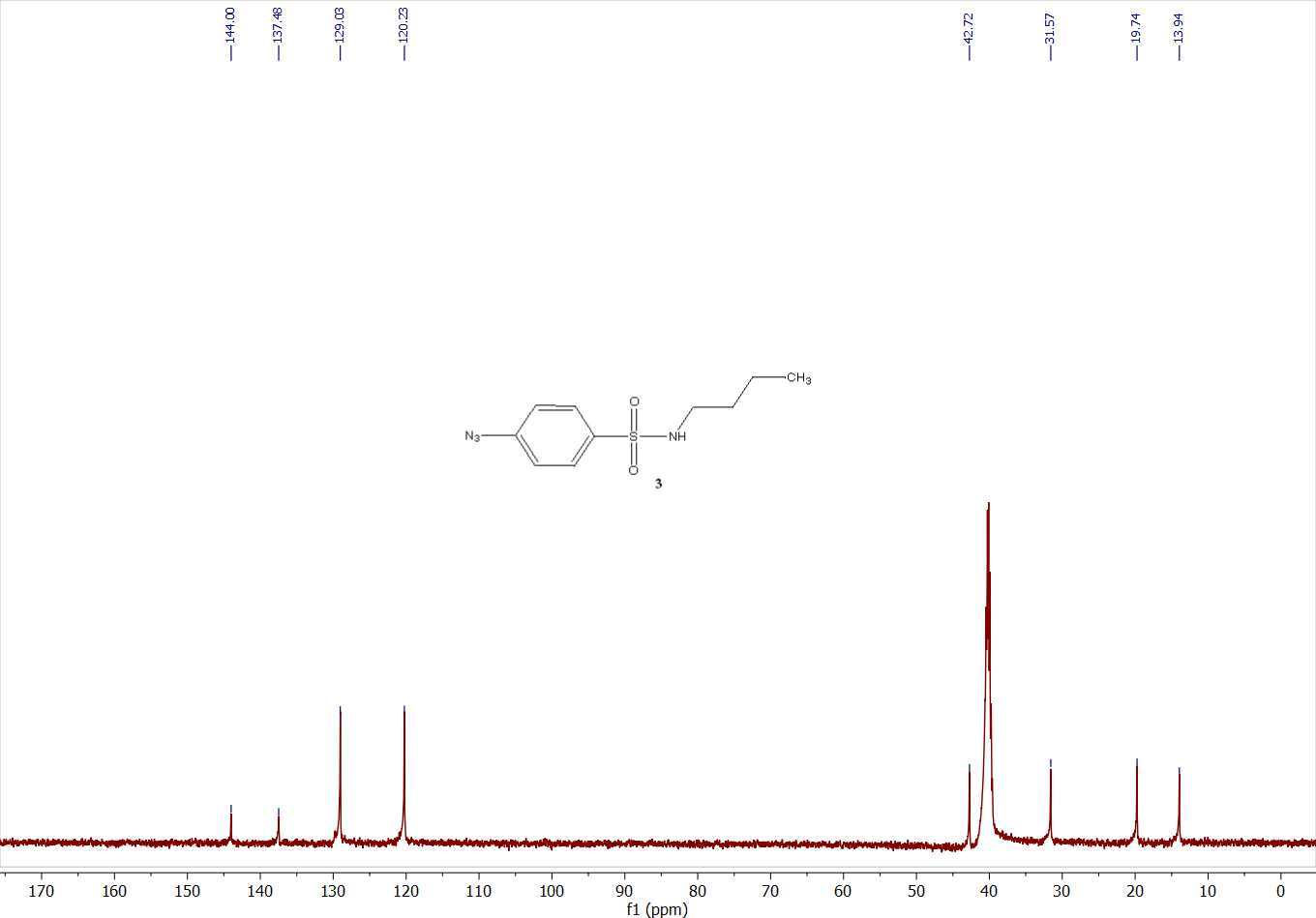
**

Figure S2. ^13^C NMR spectrum of compound **3** (DMSO-d_6_, 126 MHz, 298 K).

**
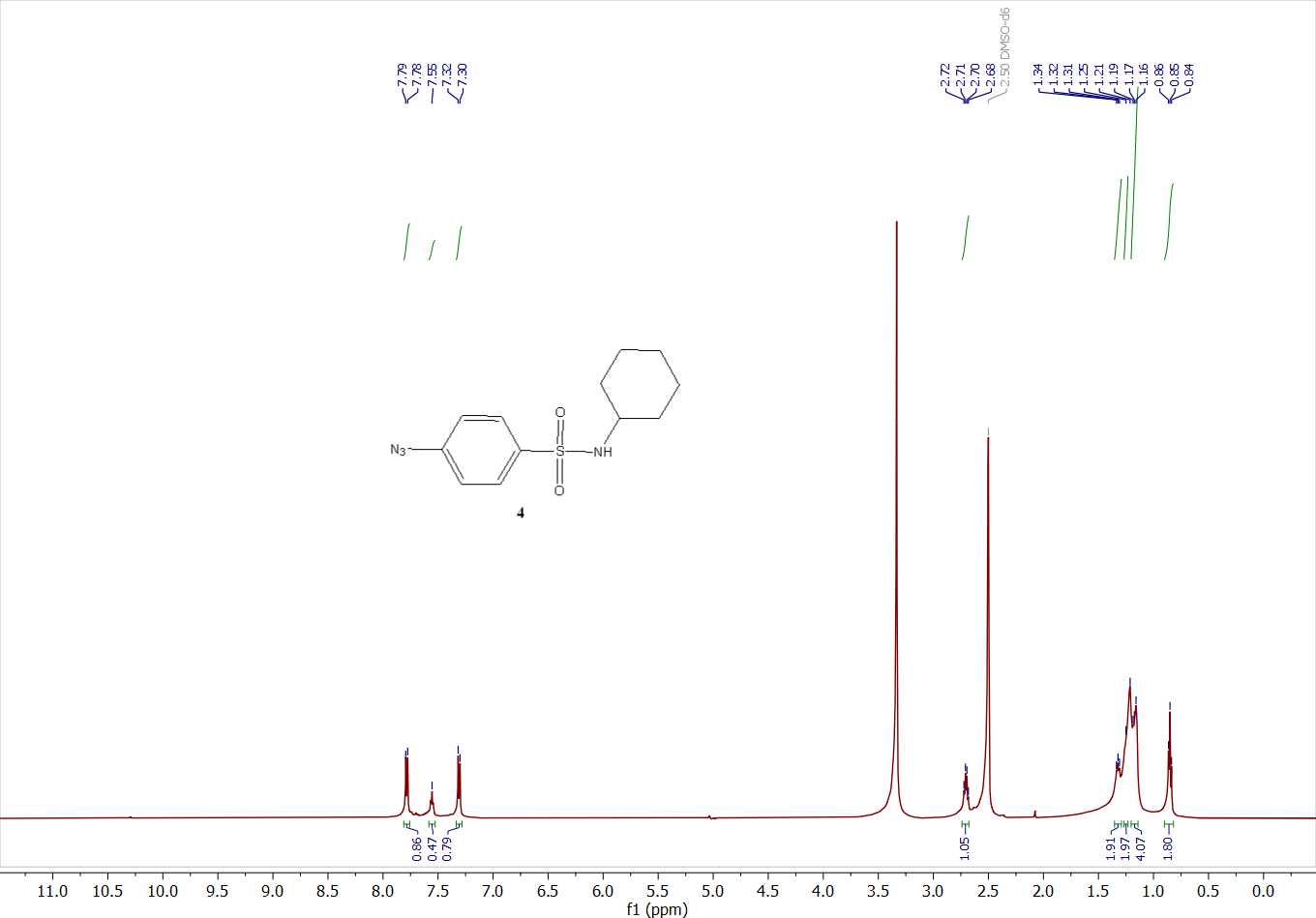
**

Figure S3. ^1^H NMR spectrum of compound **4** (DMSO-d_6_, 500 MHz, 298 K).

**
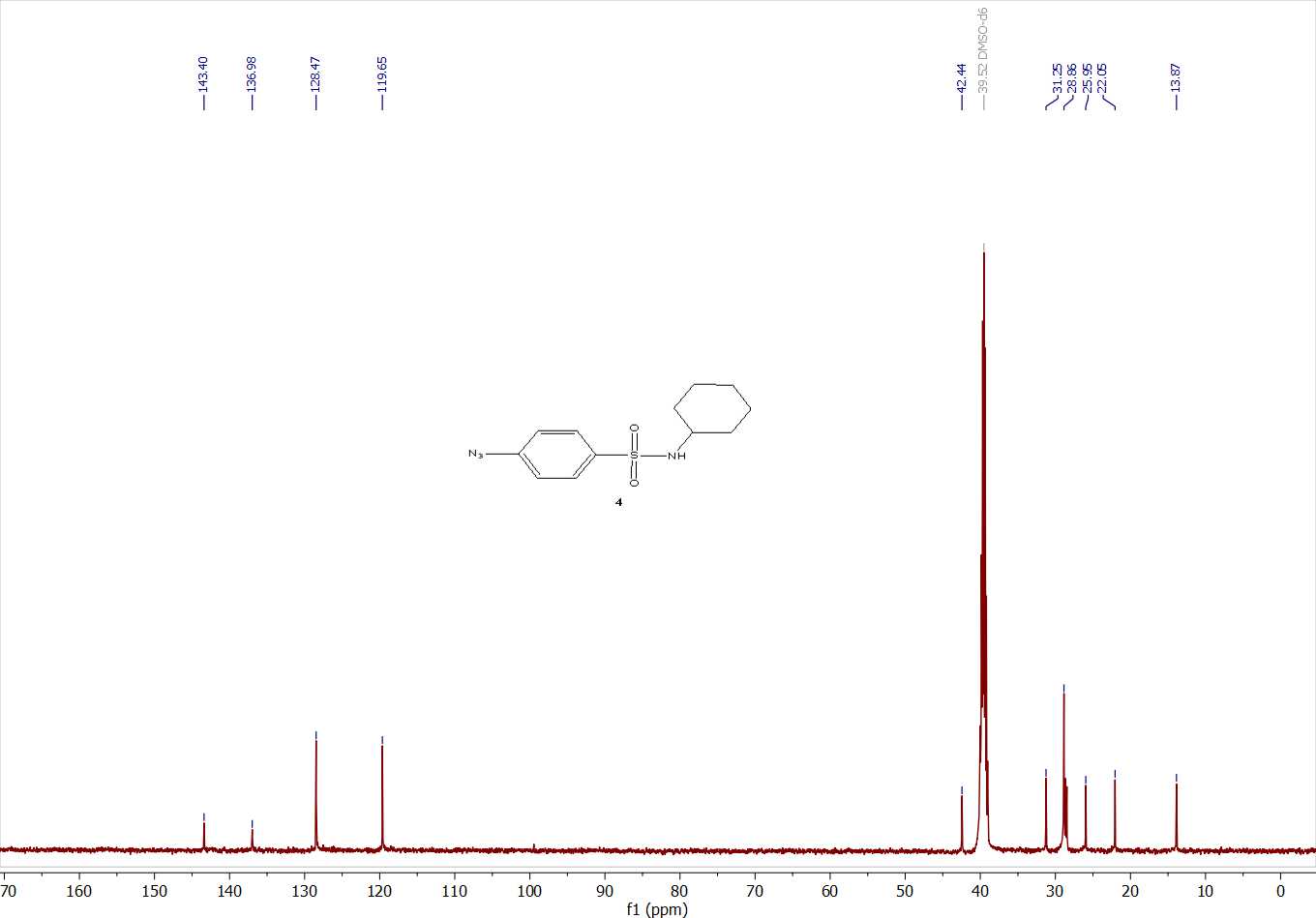
**

Figure S4. ^13^C NMR spectrum of compound **4** (DMSO-d_6_, 126 MHz, 298 K).

**
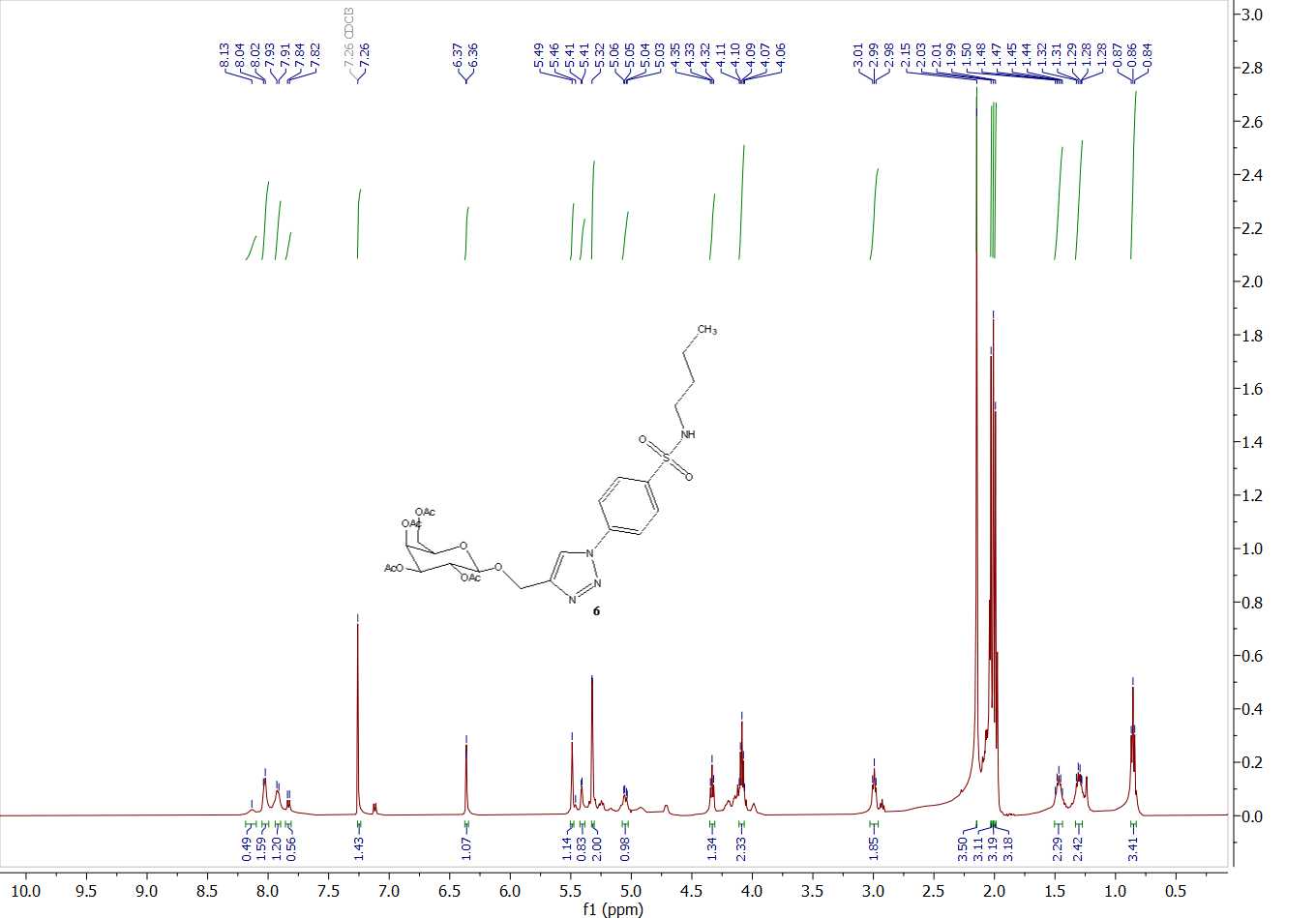
**

Figure S5. ^1^H NMR spectrum of compound **6** (CDCl3, 500 MHz, 298 K).

**
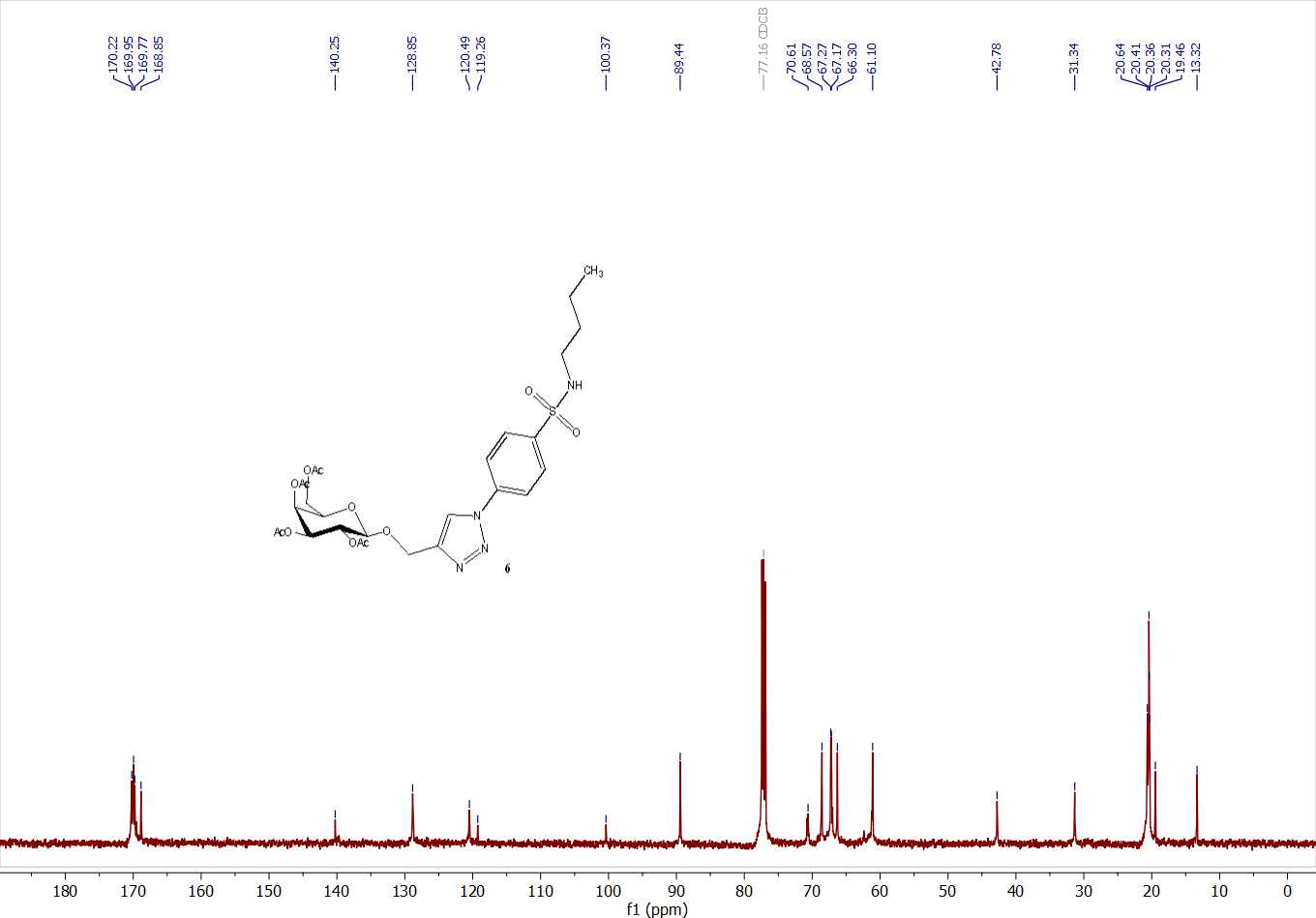
**

Figure S6. ^13^C NMR spectrum of compound **6** (CDCl3, 126 MHz, 298 K).

**
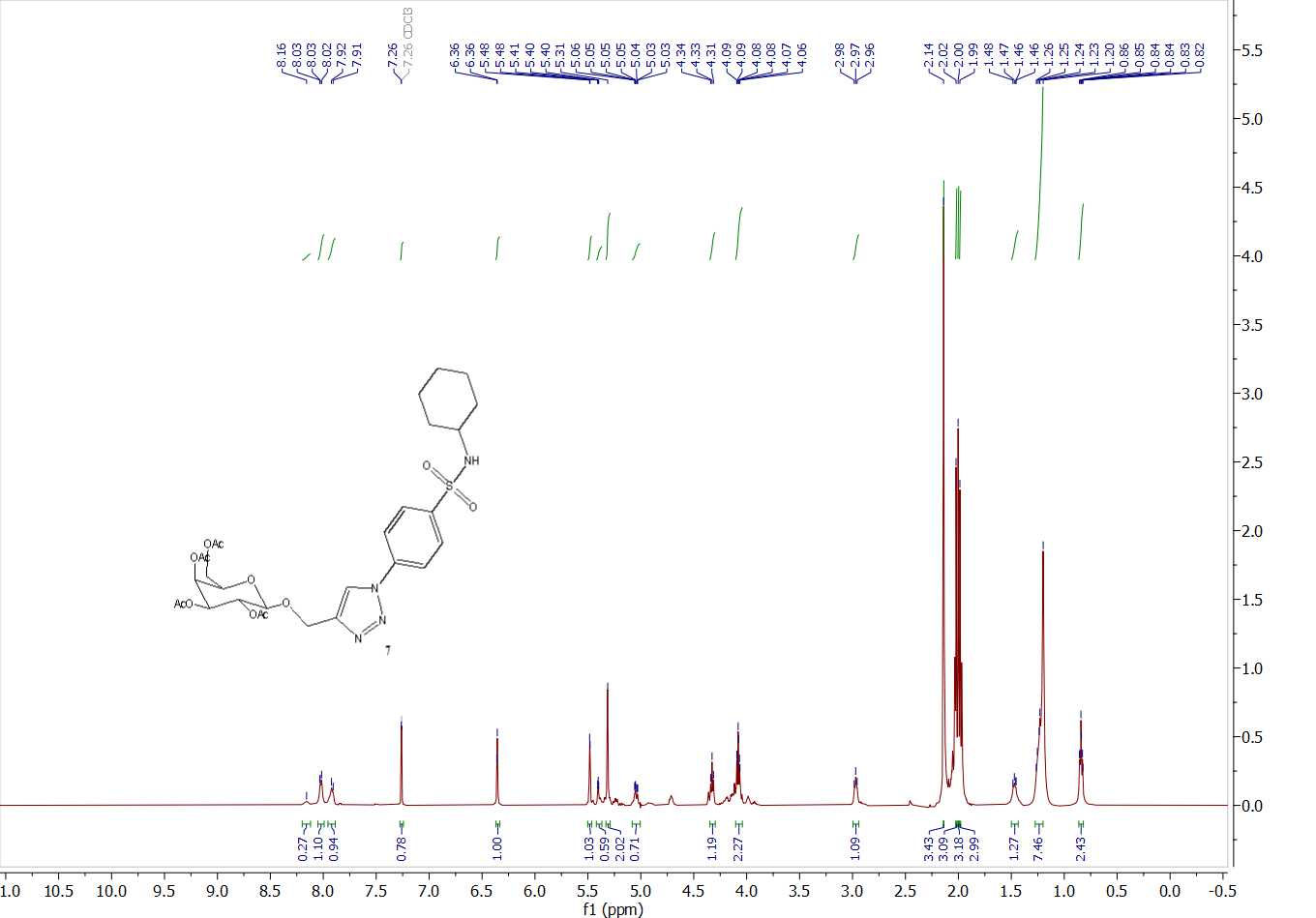
**

Figure S7. ^1^H NMR spectrum of compound **7** (CDCl3, 500 MHz, 298 K).

**
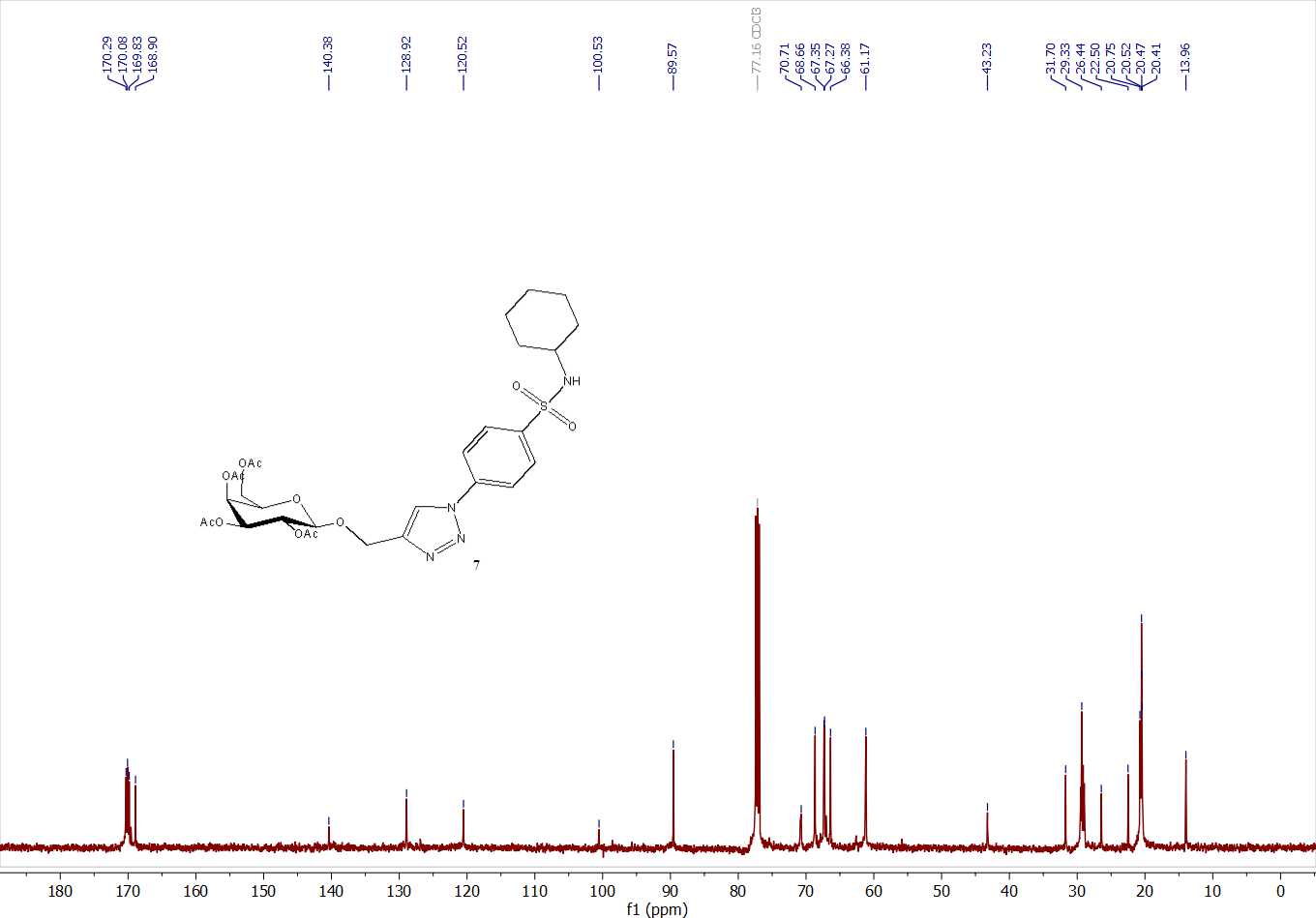
**

Figure S8. ^13^C NMR spectrum of compound **7** (CDCl3, 126 MHz, 298 K).

**
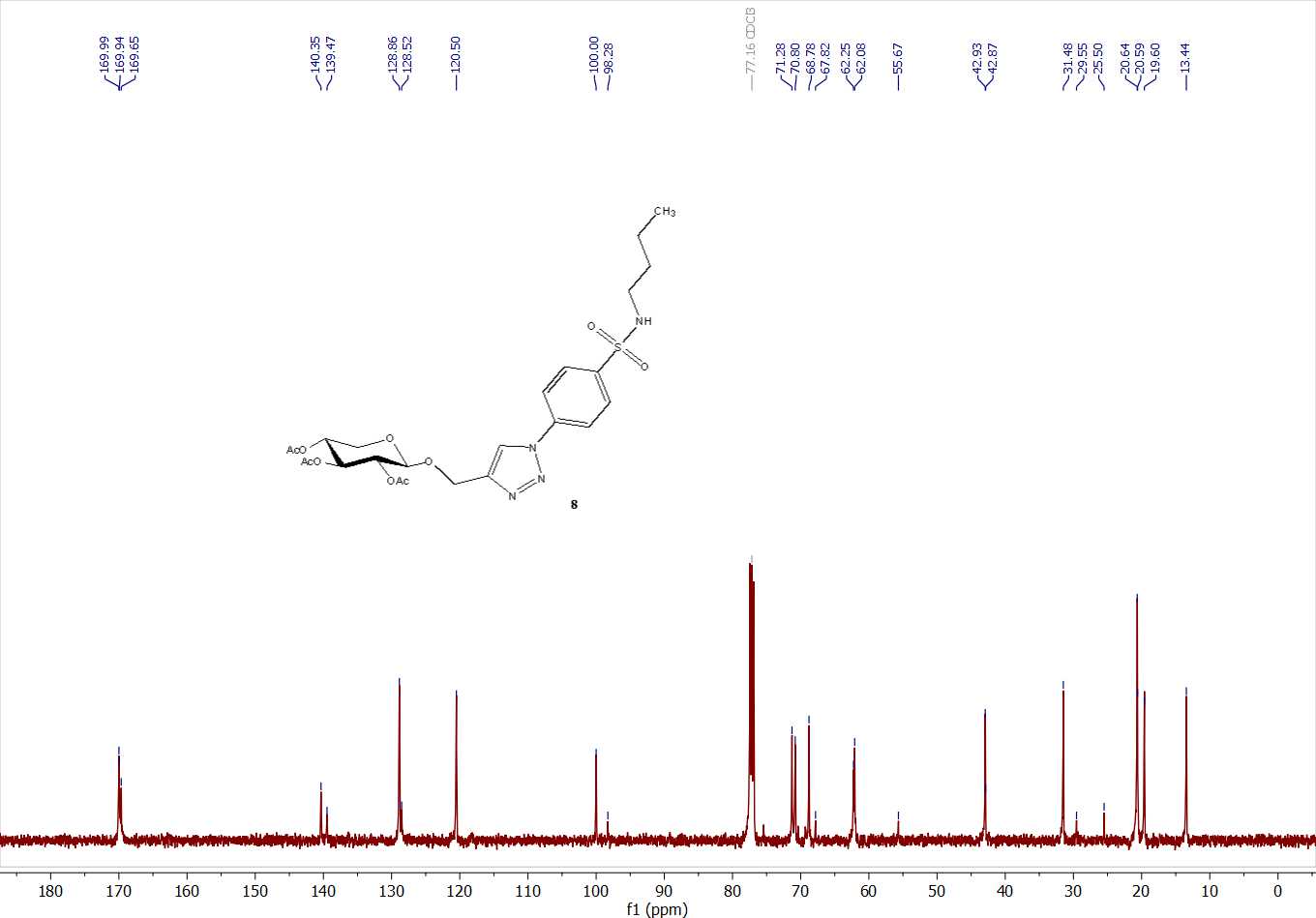
**

Figure S9. ^13^C NMR spectrum of compound **8** (CDCl3, 126 MHz, 298 K).

**
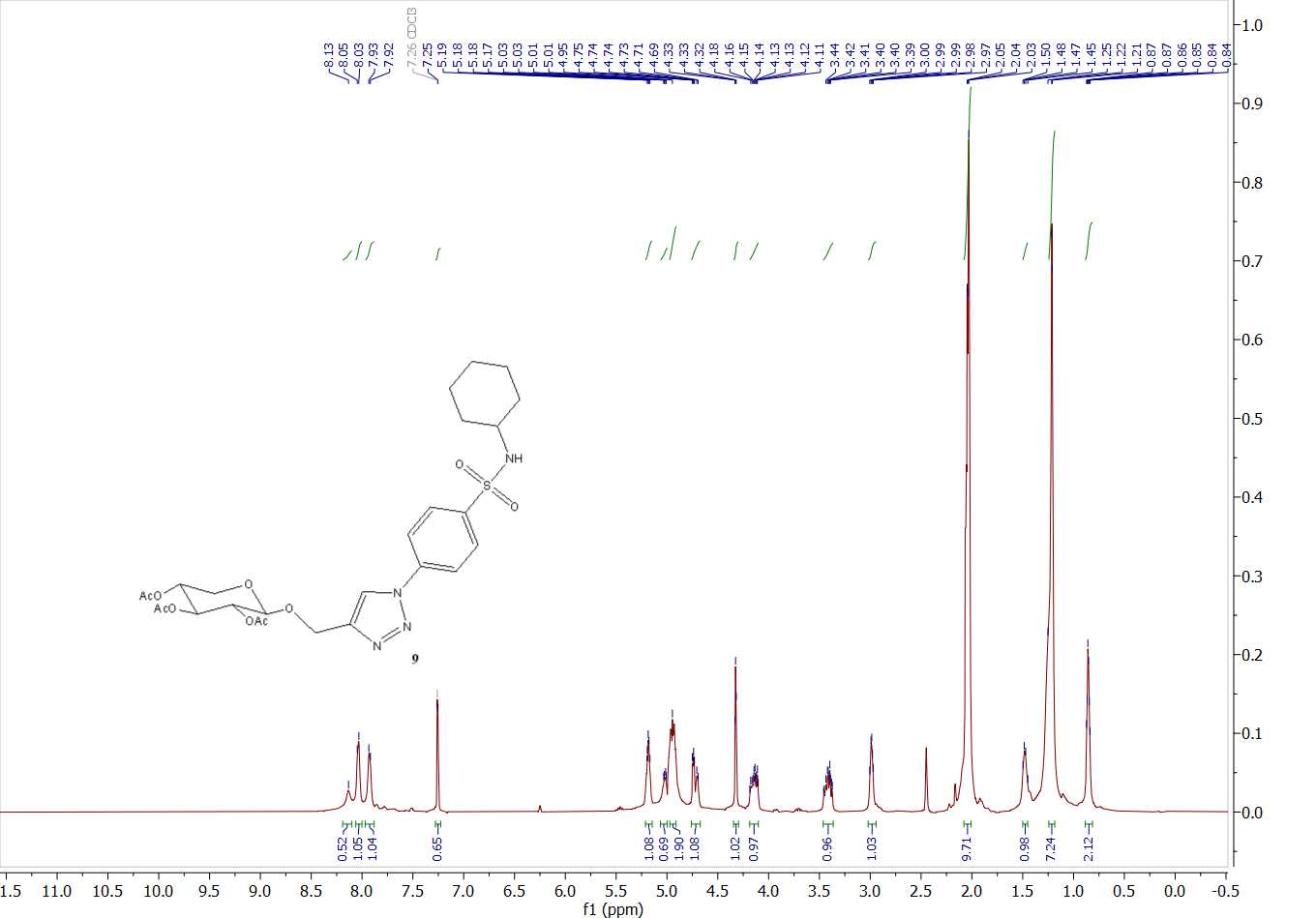
**

Figure 10. ^1^H NMR spectrum of compound **9** (CDCl3, 500 MHz, 298 K).

**
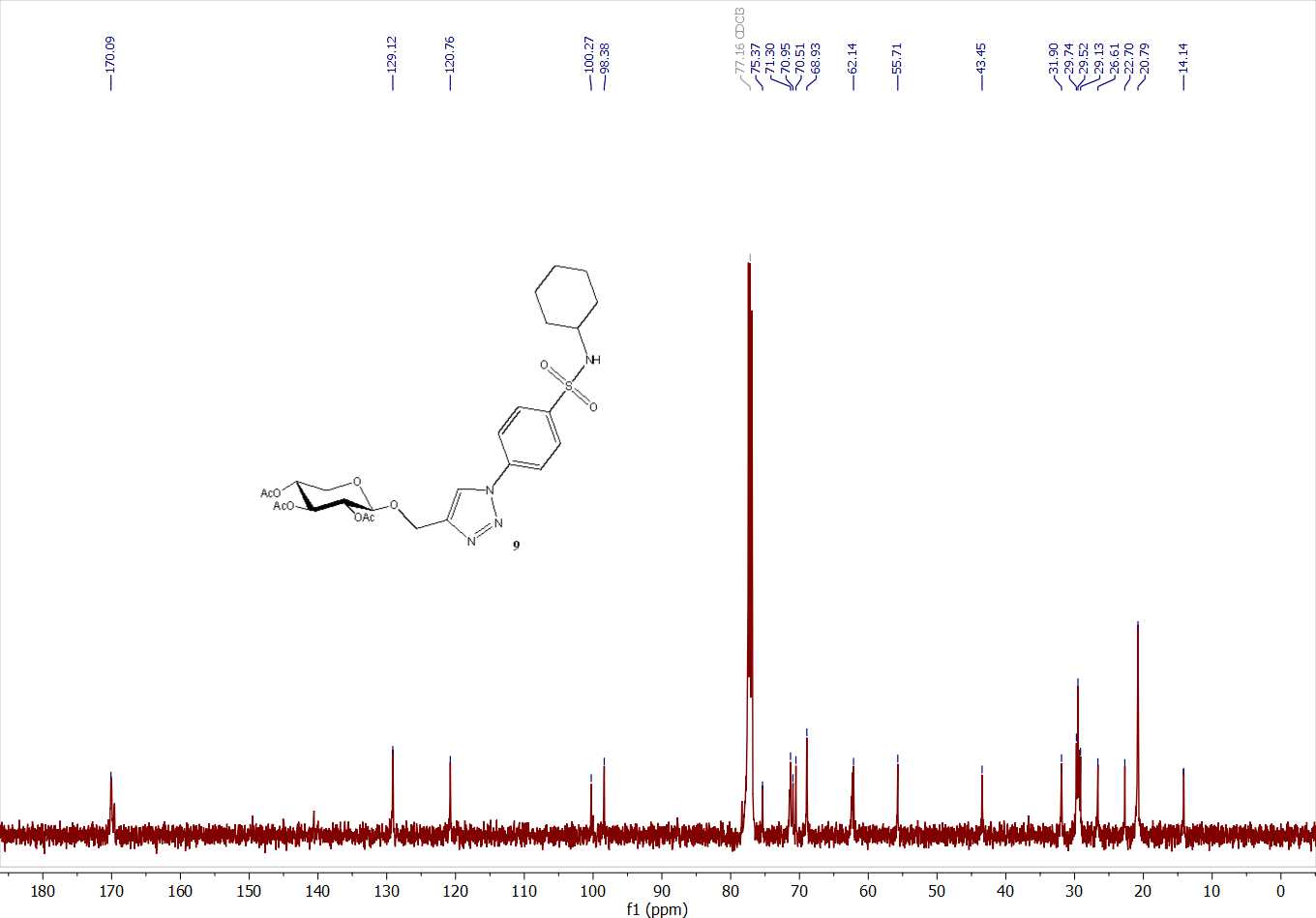
**

Figure 11. ^13^C NMR spectrum of compound **9** (CDCl3, 126 MHz, 298 K).

**
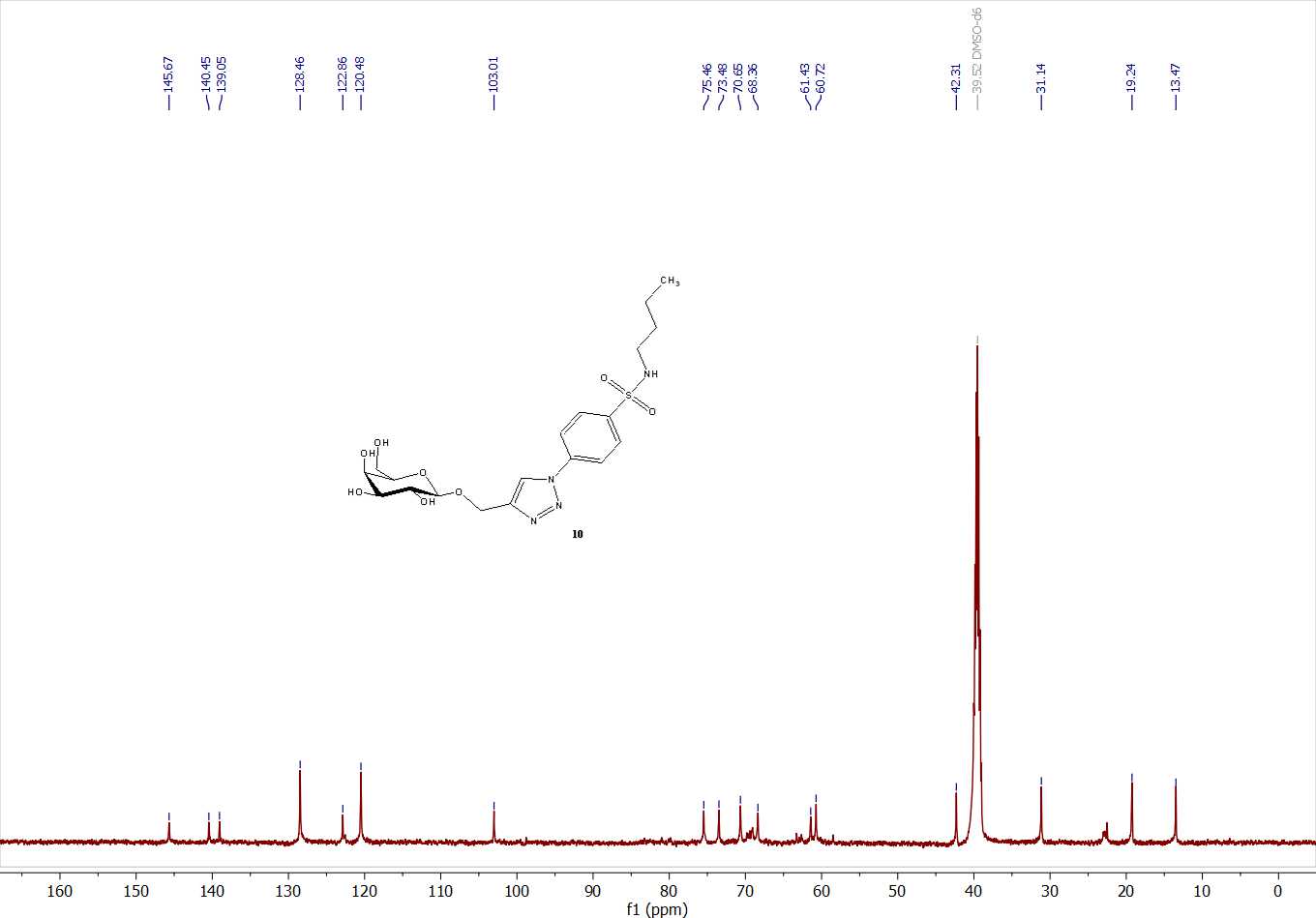
**

Figure 12. ^13^C NMR spectrum of compound **10** (DMSO-d_6_, 126 MHz, 298 K).

**
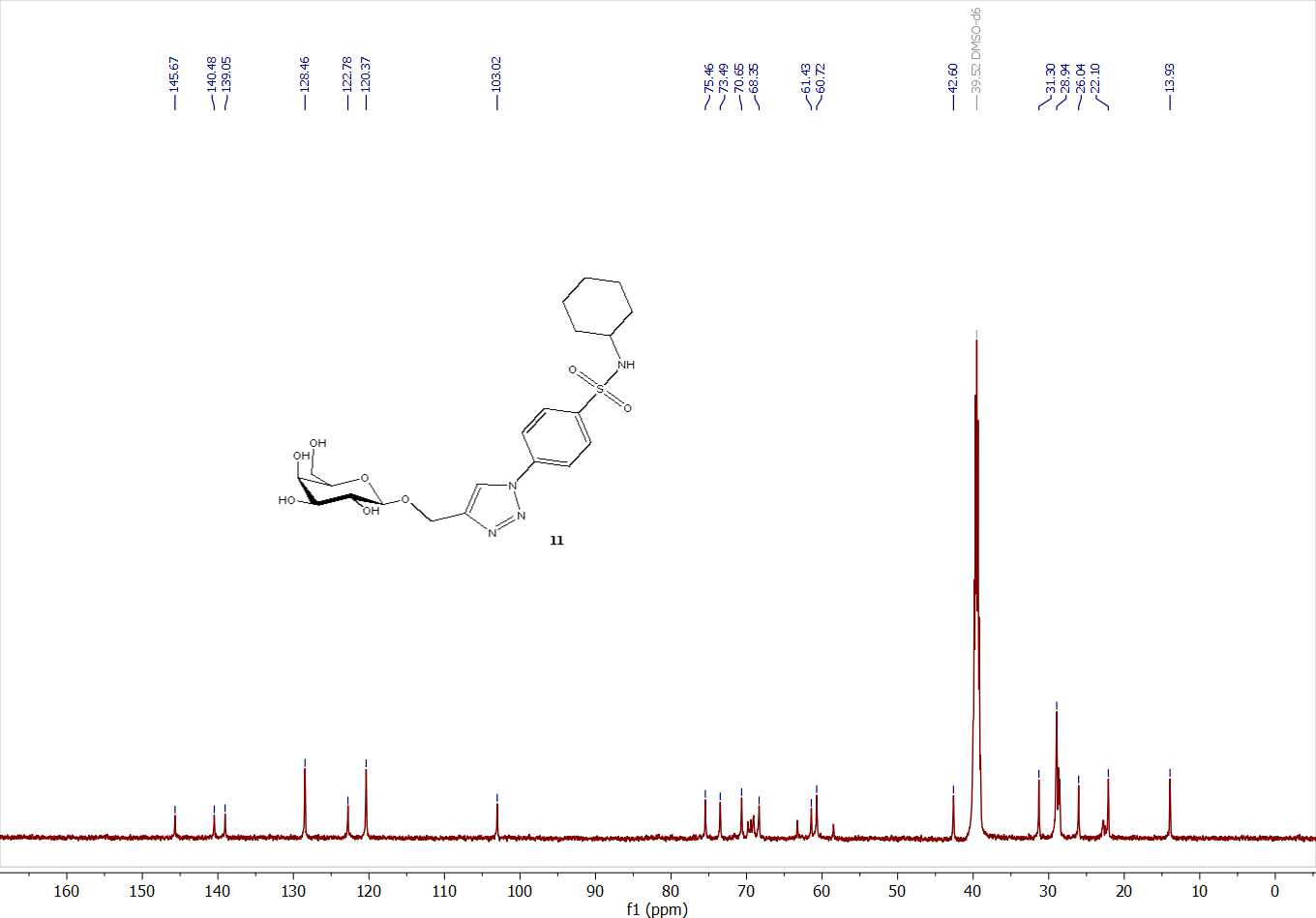
**

Figure 13. ^13^C NMR spectrum of compound **11** (DMSO-d_6_, 126 MHz, 298 K).

**
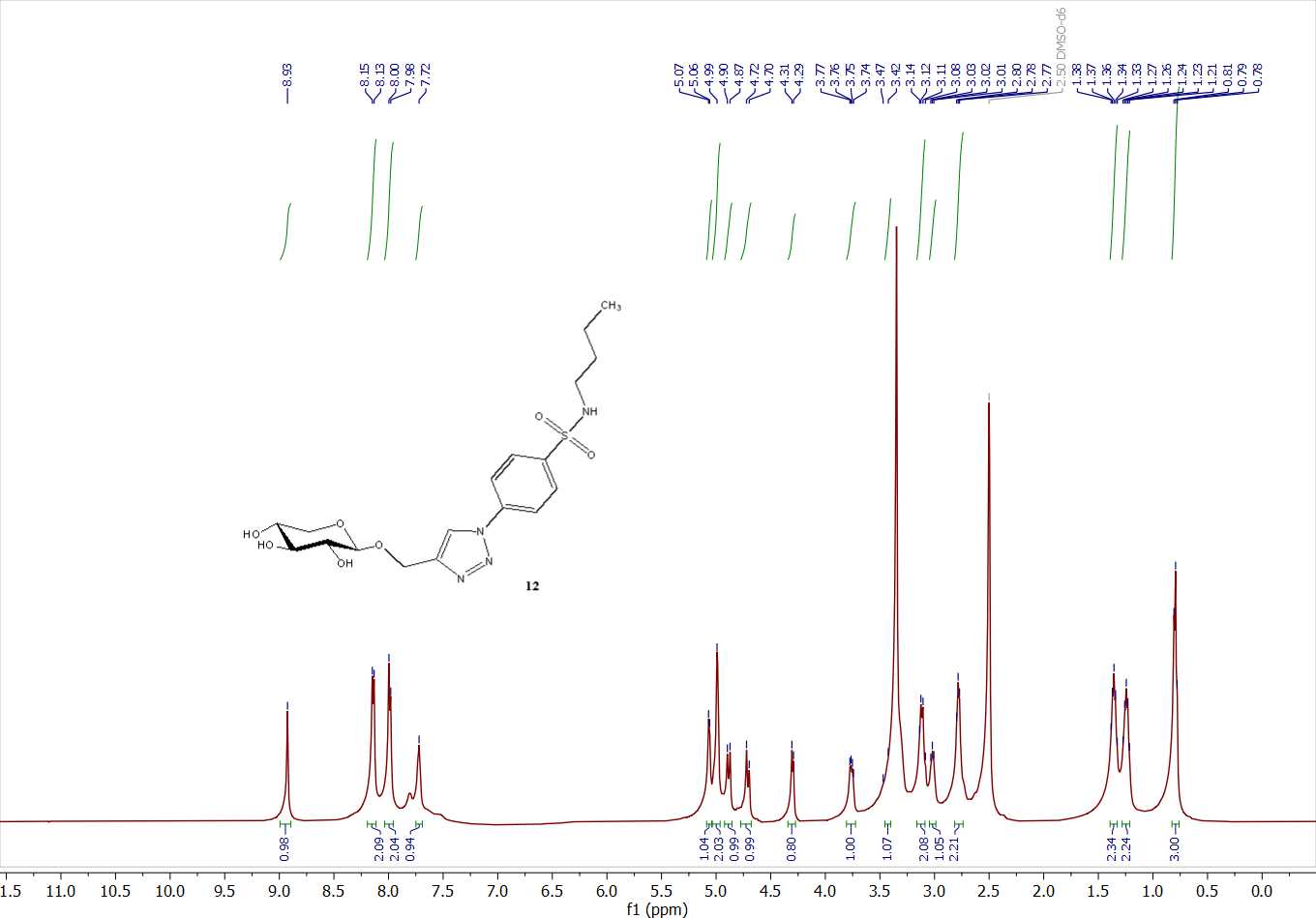
**

Figure 14. ^1^H NMR spectrum of compound **12** (DMSO-d_6_, 500 MHz, 298 K).

**
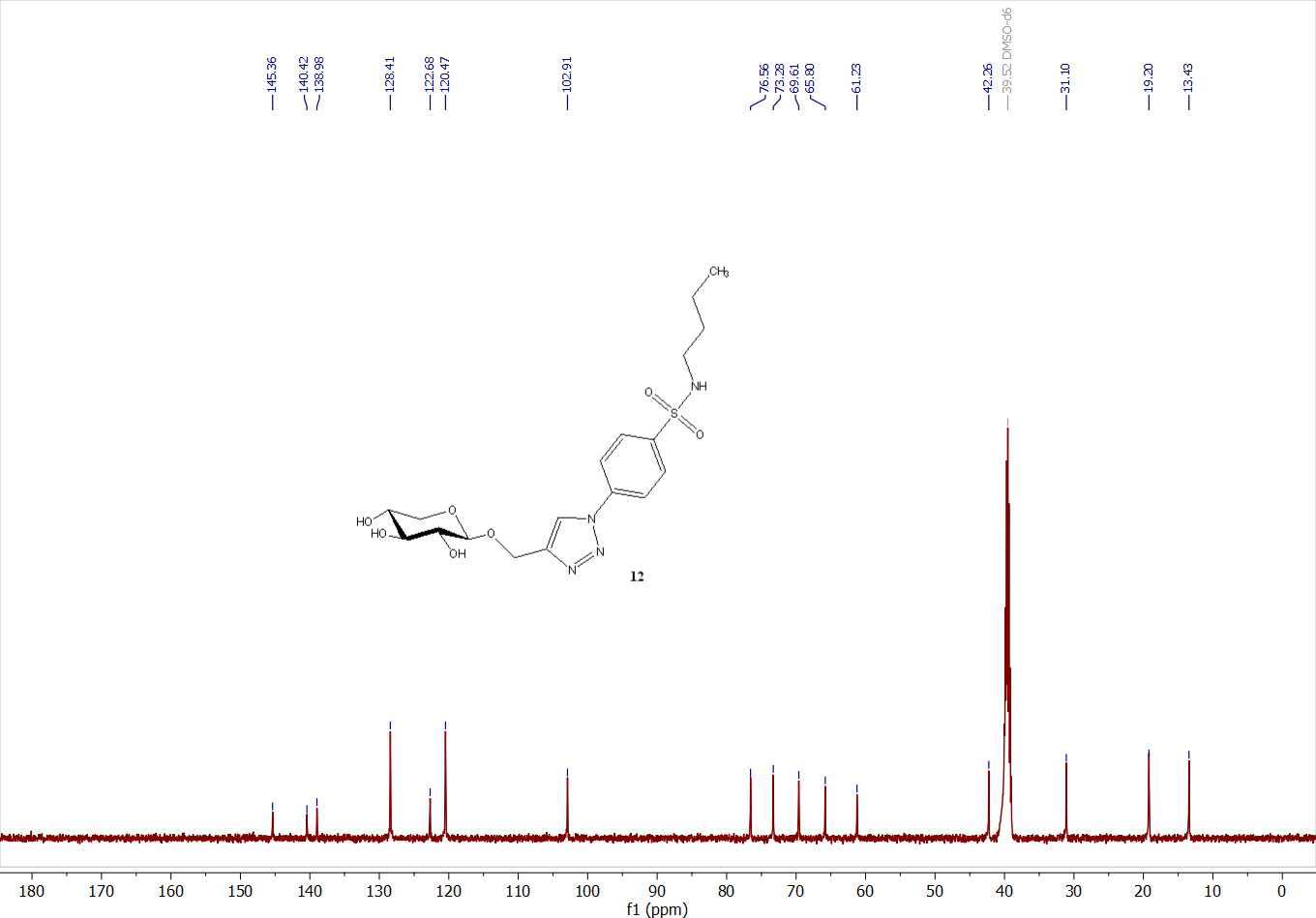
**Figure 15. ^13^C NMR spectrum of compound **12** (DMSO-d_6_, 126 MHz, 298 K).

**
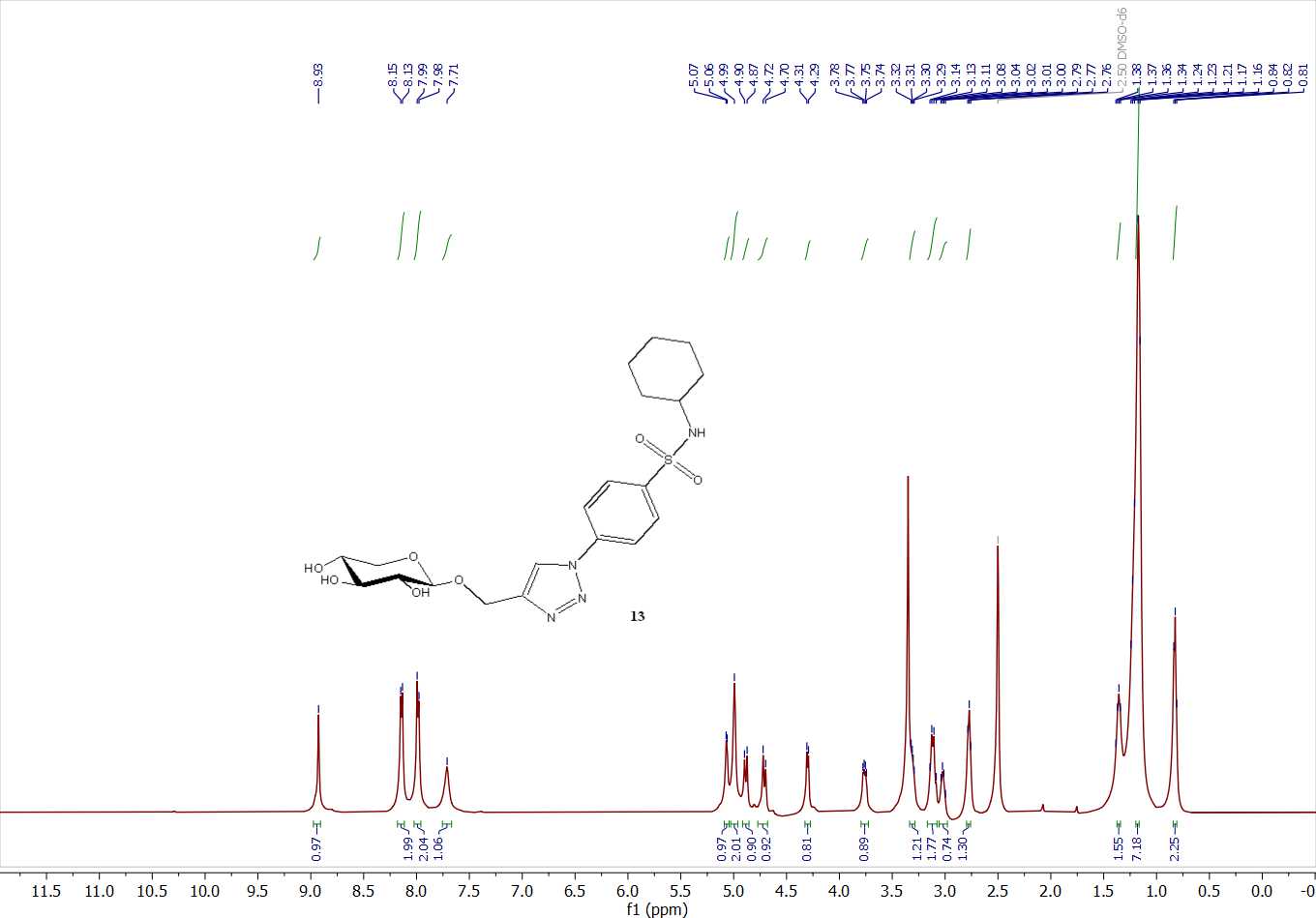
**

Figure 16. ^1^H NMR spectrum of compound **13** (DMSO-d_6_, 500 MHz, 298 K).

**
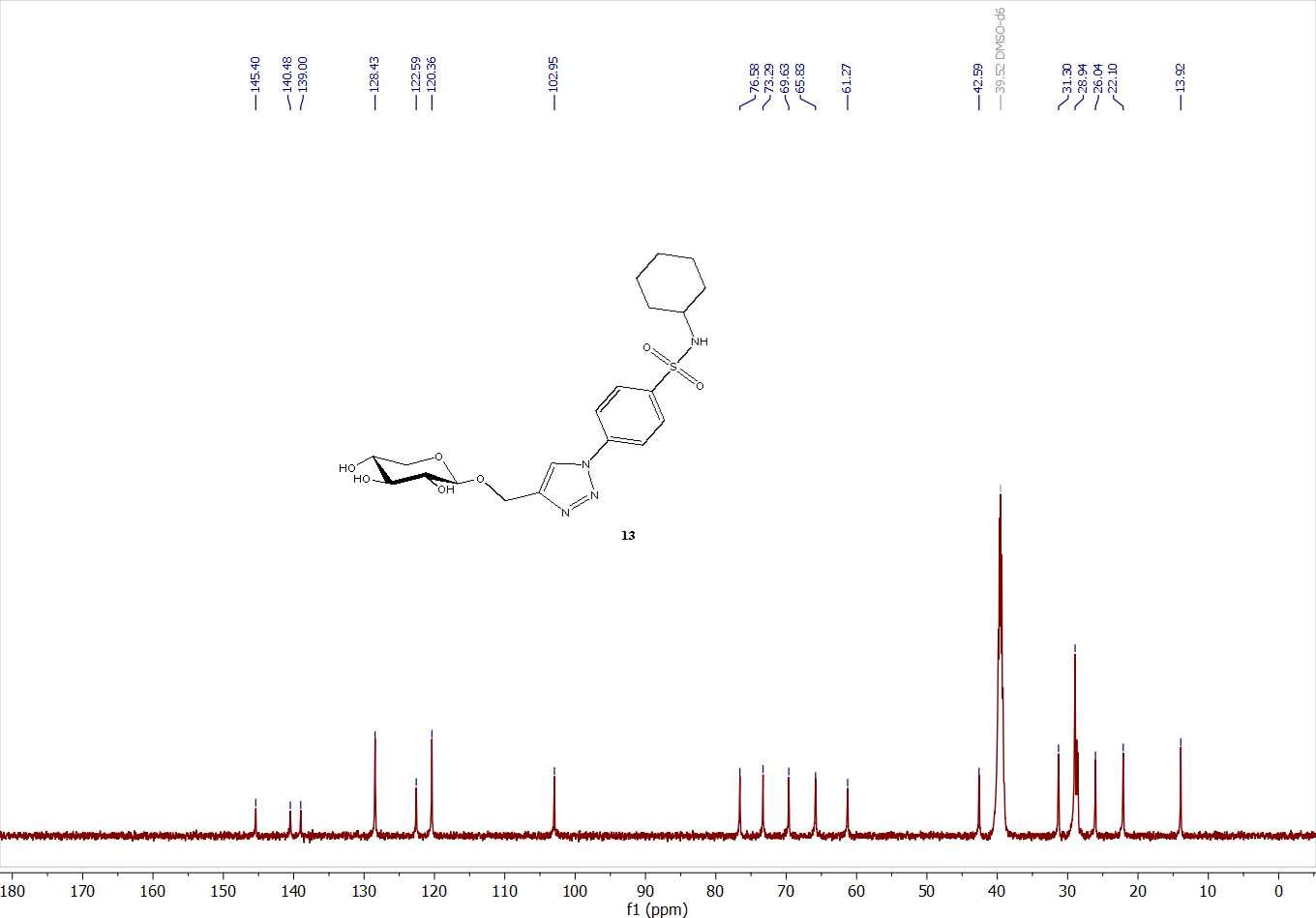
**

Figure 17. ^13^C NMR spectrum of compound **13** (DMSO-d_6_, 126 MHz, 298 K).

- 1. **Biological evaluation**
     1. ***In vitro* cytotoxic screening**

The cell lines were obtained from Karolinska Center, Department of Oncology and Pathology, Karolinska Institute and Hospital, Stockholm, Sweden. as follows: human lung A-549, liver HepG-2, breast MCF-7 and colorectal HCT-116 cancer cell lines and human retinal pigment epithelial normal RPE-1 cell line. Exponentially, cells were placed in 10^4^ cells/ well for 24 h, and then add fresh medium which containing different concentration of the tested sample. Serial two-fold dilution of the tested sample were added using a multichannel pipette. Moreover, all cells were cultivated at 37 °C, 5% CO_2_ and 95% humidity. Also, incubation of control cells occurred at 37 °C. However, after incubation for 24 h different concentrations of sample (100, 50, 25 and 12.5 µM) were added and continued the incubation for 48 h, then, add the crystal violet solution 1% to each well for 0.5 h to examine viable cells. Rinse the wells using water until no stain. After that, add 30% glacial acetic acid to all wells with shaking plates on Microplate reader (TECAN, Inc.) to measure the absorbance, using a test wavelength of 490 nm. Besides, compare the treated samples with the control cell. The cytotoxicity was estimated by IC_50_ in (μM), the concentration that inhibits 50% of growth of cancer cell.

- - 1. ***In vitro* inhibition assay of VEGFR-2 and Carbonic anhydrase isoforms hCA IX and hCA XII activities**

VEGFR-2 assay: the effect of the most promising cytotoxic compounds **4, 7** and **9** on the level of VEGFR-2 in human breast cancer cell line MCF-7 was determined. The cells in culture medium were treated with 20 μl of IC50 values of the compounds dissolved in DMSO, then incubated for 24 hours at 37 ºC, in a humidified 5% CO_2_ atmosphere. The cells were harvested and the homogenates were prepared in saline using a tight pestle homogenizer until complete cell disruption. The kit uses a double-antibody sandwich enzyme-linked immunosorbent assay (ELISA) to determine the level of human VEGFR-2 in samples. A monoclonal antibody for VEGFR-2 was pre-coated onto 96-well plates. The test samples are added to the wells and a biotinylated detection polyclonal antibody from goat specific for VEGFR-2 was added subsequently followed by washing with PBS buffer. Avidin-Biotin-Peroxidase complex was added and the unbound conjugates were washed away with PBS buffer. HRP substrate TMB was used to visualize HRP enzymatic reaction. TMB was catalyzed by HRP to produce a blue color product that changed into yellow after adding acidic stop solution. The density of yellow color is proportional to the human VEGFR-2 amount of the sample captured in the plate. The chroma of color and the concentration of the human VEGFR-2 of the samples were positively correlated and the optical density was determined at 450 nm. The level of human VEGFR-2 in samples was calculated (pg/ml) as duplicate determinations from the standard curve. Percent inhibition was calculated in comparison to control untreated cells.

An applied photophysics stopped-flow instrument was used for assaying the CA-catalyzed CO_2_ hydration activity. Phenol red (at 0.2 mM) was used as indicator, working at the absorbance maximum of 557 nm, with 20 mM Hepes (pH 7.4) and 20 mM Na_2_SO_4_ (for maintaining constant the ionic strength), following the initial rates of the CA-catalyzed CO_2_ hydration reaction for 10–100 s. The CO_2_ concentrations ranged from 1.7 to 17 mM for the determination of the kinetic parameters and inhibition constants. For each inhibitor, at least six traces of the initial 5–10% of the reaction were used for determining the initial rate. The uncatalyzed rates were determined in the same manner and subtracted from the total observed rates. Stock solutions of inhibitor (0.1 mM) were prepared in distilled-deionized water, and dilutions up to 0.01 nM were done thereafter with distilled-deionized water. Inhibitor and enzyme solutions were preincubated together for 15 min at room temperature before assay in order to allow for the formation of the E-I complex. The inhibition constants were obtained by nonlinear least-squares methods using PRISM 3, and the Cheng –Prusoff equation, and represent the mean from at least three different determinations. All CA isoforms were recombinant ones obtained in-house.

- - 1. **Cell cycle arrest and apoptosis of compound 9**

Cell cycle analysis and apoptosis study were carried out using flow cytometry. MCF-7 cells were seeded at 8×10^4^ and incubated at 37°C in 5% CO_2_ overnight. After treatment with the tested compound 10 for 24 h, cell pellets were collected and centrifuged (300 g, 5 min). For cell cycle analysis cell pellets were fixed with 70% ethanol on ice for 15 min and collected again. The pellets were incubated with propidium iodide (PI) staining solution at room temperature for 1 h and analyzed by a Gallios flow cytometer (Beckman Coulter, Brea, CA, USA). Apoptosis detection was carried out by FITC AnnexinV/PI commercial kit (Becton Dickenson, Franklin Lakes, NJ, USA) following the manufacturer protocol. The samples were analyzed by fluorescence-activated cell sorting (FACS) with a Gallios flow cytometer (Beckman Coulter, Brea, CA, USA) within 1 h after staining. Data were analyzed using Kaluza v 1.2 (Beckman Coulter).

- - 1. **Estimation the levels of p53, Bax and Bcl-2.**

The levels of the apoptotic marker Bax and anti-apoptotic marker Bcl-2 were estimated using BIO RAD iScript TM One-Step RT-PCR kit with SYBR® Green. The procedure of the used kit was done according to the manufacturer’s instructions.

Human p53 present in MCF-7 cells was determined; using Human p53 ELISA-Kit (CS0070 Sigma) read using spectrophotometer at 450 nm against untreated control cells (negative control) applying the standard protocols of the manufacturers. The samples or standard having human p53 bind to antibodies adsorbed to the microwells. Addition of biotin-conjugated was followed by incubation and addition of dispense of unbound biotin-conjugated streptavidin HRP. Then, the reaction was terminated by adding acid, and the absorbance was measured at 450 nm.

- 1. **Molecular docking study**

The molecular docking simulation of the promising *in vitro* screened benzenesulfonamide-1,2,3-triazole-glycosides **7** and **9** against VEGFR-2 and the carbonic anhydrase isoforms hCA IX and hCA XII was done using the Molecular Operating Environment software (MOE-Dock) version 2014.0901. The co-crystallized structures of VEGFR-2 and the carbonic anhydrase isoforms hCA IX and hCA XII complexed with their native ligands, sorafenib and acetazolamide were downloaded from the protein data bank (PDB codes: 4ASD, 3IAI and 1JG0, respectively). All minimizations were performed using MOE until an RMSD gradient of 0.05 kcal∙mol^−1^Å^−1^ with MMFF94x force field and the partial charges were automatically calculated. Preparation of the enzyme structures was done for molecular docking using Protonate 3D protocol with the default options in MOE. London dG scoring function and Triangle Matcher placement method were used in the docking protocol. Initially, the original ligands were re-docked into the active binding sites of VEGFR-2, hCA IX and hCA XII to assess the root-mean-square deviation values. Then, the docking studies of the newly targeted compounds were estimated within the ATP-binding sites after elimination of the co-crystallized ligands.

**Tables and figures**

**Table S1**. Preliminary antiproliferative activities of sulfonamide-based derivatives **3, 4, 6**–**13** according to the MTT assay against human cancer A-549, HepG-2, MCF-7 and HCT-116 cell lines at 100 µM for 48 h.

| Compd. No. | % inhibition ± SD | | | | |
| --- | --- | --- | --- | --- | --- |
|  | **A-549** | **HepG-2** | **MCF-7** | **HCT-116** |  |
| 3 | 20.47±0.15 | 71.40±0.91 | 51.97±0.29 | 16.70±0.60 |  |
| 4 | 97.43±0.67 | 87.30±0.91 | 91.20±0.09 | 94.83±0.87 |  |
| 6 | 32.53±0.15 | 8.93±0.74 | 37.45±0.52 | 0.00±0.05 |  |
| 7 | 94.10±0.51 | 87.60±1.73 | 98.89±0.19 | 75.70±0.96 |  |
| 8 | 58.10±0.56 | 69.17±0.21 | 66.04±0.69 | 41.05±0.18 |  |
| 9 | 90.00±0.95 | 82.60±1.73 | 92.73±1.04 | 75.70±0.48 |  |
| 10 | 32.03±0.84 | 35.85±0.42 | 25.35±0.91 | 8.91±2.70 |  |
| 11 | 30.14±0.62 | 73.85±1.06 | 20.83±0.79 | 39.00±0.96 |  |
| 12 | 17.83±0.95 | 62.86±1.19 | 41.37±0.91 | 0.00±0.005 |  |
| 13 | 54.63±0.97 | 75.10±0.12 | 71.43±0.03 | 70.10±1.05 |  |
| Doxorubicin | 100±0.20 | 99.60±0. 16 | 100±0.08 | 100±0.25 |  |

Each result is a mean of three replicate samples and values are represented as % inhibition, ± represents standard deviation.

**Table S2.** The percentage cytotoxicity of the active compounds **4**, **7** and **9** on A-549 human tumor cell line at different concentrations

| **Compounds** | **100 µM** | **50 µM** | **25 µM** | **12.5 µM** |
| --- | --- | --- | --- | --- |
| **4** | 97.43±0.67 | 70.87±1.63 | 34.87±0.75 | 21.37±1.55 |
| **7** | 94.10±0.51 | 60.23±1.47 | 29.31±0.02 | 12.60±0.82 |
| **9** | 90.00±0.95 | 75.53±1.16 | 47.60±0.32 | 43.13±0.73 |

*The results are shown as average ± standard deviation.

**Table S3.** The percentage cytotoxicity of the active compounds **3**, **4**, **7**-**9**, **11**-**13** on HepG-2 human tumor cell line at different concentrations

| **Compounds** | **100 µM** | **50 µM** | **25 µM** | **12.5 µM** |
| --- | --- | --- | --- | --- |
| **3** | 71.40±0.91 | 49.40±1.91 | 19.08±1.88 | 3.76±5.32 |
| **4** | 87.30±0.91 | 85.67±2.50 | 86.23±0.98 | 85.13±1.65 |
| **7** | 87.60±1.73 | 85.58±0.88 | 85.07±0.05 | 84.09±0.85 |
| **8** | 69.17±0.21 | 50.73±1.45 | 32.80±2.12 | 23.15±0.43 |
| **9** | 82.60±1.73 | 82.59±0.87 | 63.70±2.29 | 58.20±1.50 |
| **11** | 73.85±1.06 | 62.70±0.36 | 57.70±0.87 | 44.80±0.50 |
| **12** | 62.86±1.19 | 55.95±0.44 | 44.70±1.13 | 33.95±0.33 |
| **13** | 75.10±0.12 | 56.20±1.29 | 38.80±0.40 | 43.30±0.66 |

*The results are shown as average ± standard deviation.

**Table S4.** The percentage cytotoxicity of the active compounds **4**, **7**, **8**, **9** and **13** on MCF-7 human tumor cell line at different concentrations

| **Compounds** | **100µM** | **50µM** | **25µM** | **12.5µM** |
| --- | --- | --- | --- | --- |
| **4** | 91.20±0.09 | 78.50±0.37 | 58.31±3.30 | 35.03±2.83 |
| **7** | 98.89±0.19 | 88.77±2.70 | 55.90±1.44 | 30.37±3.66 |
| **8** | 66.04±0.69 | 49.80±0.41 | 36.13±2.06 | 29.56±1.66 |
| **9** | 92.73±1.04 | 83.30±1.87 | 52.27±0.43 | 32.60±6.13 |
| **13** | 71.43±2.03 | 54.73±1.88 | 48.07±4.31 | 34.87±5.91 |

*The results are shown as average ± standard deviation.

**Table S5.** The percentage cytotoxicity of the active compounds **4**, **7**, **9** and **13** on HCT-116 human tumor cell line at different concentrations

| **Compounds** | **100 µM** | **50 µM** | **25 µM** | **12.5 µM** |
| --- | --- | --- | --- | --- |
| **4** | 94.83±0.87 | 86.90±0.28 | 51.93±0.58 | 34.64±0.48 |
| **7** | 75.70±0.96 | 50.70±0.70 | 48.80±0.95 | 32.50±1.25 |
| **9** | 75.70±0.48 | 67.00±0.12 | 53.50±0.93 | 34.51±0.44 |
| **13** | 70.10±1.05 | 47.00±0.79 | 28.51±0.69 | 11.00±0.65 |

*The results are shown as average ± standard deviation.

**Table S6.** Cell cycle analysis after 48 h incubation with compound **9**

| **Compound No.** | **%G0-G1** | **%S** | **%G2/M** |
| --- | --- | --- | --- |
| **9** /**MCF-7** | 52.69 | 17.33 | 29.98 |
| **cont./MCF-7** | 66.82 | 22.79 | 10.39 |

**Table S7.** Apoptosis induction analysis within MCF-7 cells treated with compound **9**

|  | Apoptosis | | | Necrosis |
| --- | --- | --- | --- | --- |
|  | **Total** | **Early** | **Late** |  |
| 9/ MCF-7 | 29.51 | 15.77 | 9.75 | 3.99 |
| Cont. / MCF-7 | 1.76 | 0.35 | 0.14 | 1.27 |
